# Different viral effectors hijack TCP17, a key transcription factor for host Auxin synthesis, to promote viral infection

**DOI:** 10.1371/journal.ppat.1012510

**Published:** 2024-08-29

**Authors:** Yanxiao Zhao, Yong He, Xinyue Chen, Ninghong Li, Tongqing Yang, Tingting Hu, Shujing Duan, Xuanjie Luo, Lei Jiang, Xiaoyang Chen, Xiaorong Tao, Jing Chen

**Affiliations:** 1 School of Plant Protection, Anhui Agricultural University, Hefei, China; 2 The Key Laboratory of Plant Immunity, Department of Plant Pathology, Nanjing Agricultural University, Nanjing, China; 3 Anhui Province Key Laboratory of Crop Integrated Pest Management, Anhui Agricultural University, Hefei, China; The Ohio State University, UNITED STATES OF AMERICA

## Abstract

Auxin is an important class of plant hormones that play an important role in plant growth development, biotic stress response, and viruses often suppress host plant auxin levels to promote infection. However, previous research on auxin-mediated disease resistance has focused mainly on signaling pathway, and the molecular mechanisms of how pathogenic proteins manipulate the biosynthetic pathway of auxin remain poorly understood. TCP is a class of plant-specific transcription factors, of which TCP17 is a member that binds to the promoter of *YUCCAs*, a key rate-limiting enzyme for auxin synthesis, and promotes the expression of *YUCCAs*, which is involved in auxin synthesis in plants. In this study, we reported that Tomato spotted wilt virus (TSWV) infection suppressed the expression of *YUCCAs* through its interaction with TCP17. Further studies revealed that the NSs protein encoded by TSWV disrupts the dimerization of TCP17, thereby inhibit its transcriptional activation ability and reducing the auxin content in plants. Consequently, this interference inhibits the auxin response signal and promotes the TSWV infection. Transgenic plants overexpressing TCP17 exhibit resistance against TSWV infection, whereas plants knocking out *TCP17* were more susceptible to TSWV infection. Additionally, proteins encoded by other RNA viruses (BSMV, RSV and TBSV) can also interact with TCP17 and interfere with its dimerization. Notably, overexpression of TCP17 enhanced resistance against BSMV. This suggests that TCP17 plays a crucial role in plant defense against different types of plant viruses that use viral proteins to target this key component of auxin synthesis and promote infection.

## Author summary

Tomato spotted wilt virus (TSWV) poses a significant threat to global agricultural production and is transmitted by thrips. This study discovered that TSWV can disrupt key factors in the plant auxin signaling pathway, thereby inhibiting the synthesis and signal transduction of auxin, and promoting viral invasion. Auxin is not only a crucial hormone for plant growth and development but also plays a vital role in the plant resistance pathways. TSWV infection leads to a decrease in auxin levels, affecting the plant defense mechanisms. Furthermore, the study found that other viruses, such as Barley stripe mosaic virus, can also use the same strategy to enhance their own infection, indicating that the inhibition of plant auxin synthesis and signal transduction pathways is a conserved process across different viral infections. This discovery provides new insights into the pathogenic mechanisms of plant viruses and the study of virus-host interaction and offers new ideas for the development of novel plant virus control strategies.

## Introduction

Tomato spotted wilt virus (TSWV) is the type species of Tospovirus in the family *Bunyaviridae*, the only genus of plant-infecting viruses [1,2]. TSWV, transmitted by thrips, is globally distributed and poses a significant threat to agriculture, particularly affecting tomato and pepper crops [3,4]. It induces typical symptoms such as concentric rings of spots on leaves or fruits, plant dwarfing, leaf distortion, and in severe cases, plant mortality. Therefore, the study of the pathogenic mechanism of TSWV has important economic and social benefits. The NSs protein, encoded by TWSV, serves as a silencing suppressor and an important virulence factor, playing a pivotal role in virus infection and pathogenesis [5–7].

Auxin, also known as indole-3-acetic acid (IAA), is one of the most abundant and biologically significant hormones in plants, and is widely involved in the regulation of plant growth and development [8]. Hormone synthesis is mainly derived from tryptophan metabolism dependent on the key enzymes of auxin synthesis, YUCCAs (YUCs) [9]. IAA, an endogenous molecule, directly activates auxin signaling [10,11]. This signaling is primarily perceived through the TIR1/AFB receptor with the auxin/indoleacetic acid (Aux/IAA) family of proteins [12–14]. In the presence of IAA, the TIR1 receptor binds to IAA, targeting the transcriptional repressor Aux/IAA for degradation. This degradation releases the auxin response from the transcriptionally active factor (ARF) family members, activating downstream auxin response signaling [15,16]. Notably, auxin signaling response plays an important role in plant disease resistance. For example, auxin-regulated downstream disease resistance response can prevent infections of Tobacco mosaic virus (TMV) and Rice dwarf virus (RDV) [17,18]. However, viruses are often exploit the auxin signaling pathway to facilitate their own infection [19]. RSV P2 and RBSDV SP8 target the auxin-responsive transcription factor OsARF17, disrupting its dimerization and DNA-binding activity, thereby interfering with auxin signaling and promoting viral infection [20]. The P2 coat protein and the Aux/IAA family protein OsIAA10, a repressor and co-receptor of the auxin pathway in rice, obstruct the auxin response, enhancing virus infection. Tomato chlorotic virus (ToCV) encodes a major pathogenic protein, p22, which interferes with the SCF^TIR1^ complex assembly by binding to the C-terminal end of the host protein SKP1.1,inhibiting the degradation of the auxin signaling pathway repressor, Aux/IAA, and blocking auxin signaling, thus facilitating the ToCV pathogenicity [21]. It has also been reported that auxin levels in plants fluctuate with virus infection. For example, significant changes in host root growth IAA levels were noted during Cucumber mosaic virus (CMV) infection [22], and auxin levels rose in rice following RDV infection, and exogenous auxin treatment could enhance rice resistance to RDV [23]. However, the molecular mechanisms underlying the changes in auxin content during viral infection remain largely unknown.

TCPs, a class of plant-specific transcriptional regulatory proteins with conserved TCP domains, are widely involved in plant growth and development [24–26]. They are classified into class I and class II TCP transcription factors based on their DNA binding sequences [25,27,28]. Recent studies have increasingly revealed the role of TCP transcription factors in regulating various plant hormones and participating in diverse hormone signaling pathways. For instance, TCP1 binds to DWARF4 (AtDWF4), a key enzyme in the synthesis of bilobalide (BR), promoting AtDWF4 expression and increasing endogenous BR content, which in turn supports plant growth and development [29]. TCP14 and TCP15 mediate the promotion of gibberellin on seed germination in *Arabidopsis thaliana* [30]. TCP17 and its two closely related homologs, TCP5 and TCP13, up-regulate the auxin biosynthesis and mediate shade-induced hypocotyls growths in plants through the PHYTOCHROME (PIFs) and YUCCAs pathways [31]. AtTCP18, AtTCP3 and AtTCP15 are involved in auxin signaling [32–35]. TCP proteins may also engage in various hormonal pathways such as abscisic acid (ABA), gibberellic acid (GA), and salicylic acid (SA) [36,37]. Conversely, plant pathogens manipulate phytohormone signaling pathways by attacking TCP transcription factors to promote infection. For example, Rice ragged stunt virus (RRSV) infection suppresses OsTCP21 gene expression and inhibits the jasmonic acid (JA) response pathway in rice [38]. *Pseudomonas syringae* promotes the degradation of endogenous TCP14 proteins in plants, activates the JA response pathway, and facilitates the infection of bacterial pathogen [39].

Our previous research demonstrated a significant reduction in auxin (IAA) content in TSWV-infested hosts and that the TSWV-encoded NSs protein markedly suppressed the auxin signaling response pathway [40]. However, the molecular mechanisms underlying the auxin content changes in TSWV-infected hosts have remained elusive. In this study, we report that TSWV-encoded NSs protein can disrupt with the dimerization of TCP17 and inhibit its transcriptional activation. This disruption leads to a reduction in auxin content, dependent on the synthesis of key synthases YUCs, and inhibits the auxin signaling response pathway, thereby promoting viral infection. Additionally, effectors from different RNA viruses, such as Barley stripe mosaic virus (BSMV) γb and Rice stripe virus (RSV) NS3, can interact with TCP17 and interfere with its dimerization. Overexpression of TCP17 in *Nicotiana benthamiana* enhances resistance to BSMV. Our findings provide insight into the role of the TCP17 protein in plant-virus interactions, suggesting the potential of TCP17, a key component of auxin synthesis, as a novel target for the development of strategies against various plant viruses.

## Results

### NSs inhibits auxin biosynthesis and signaling pathway

**Fig 1:**
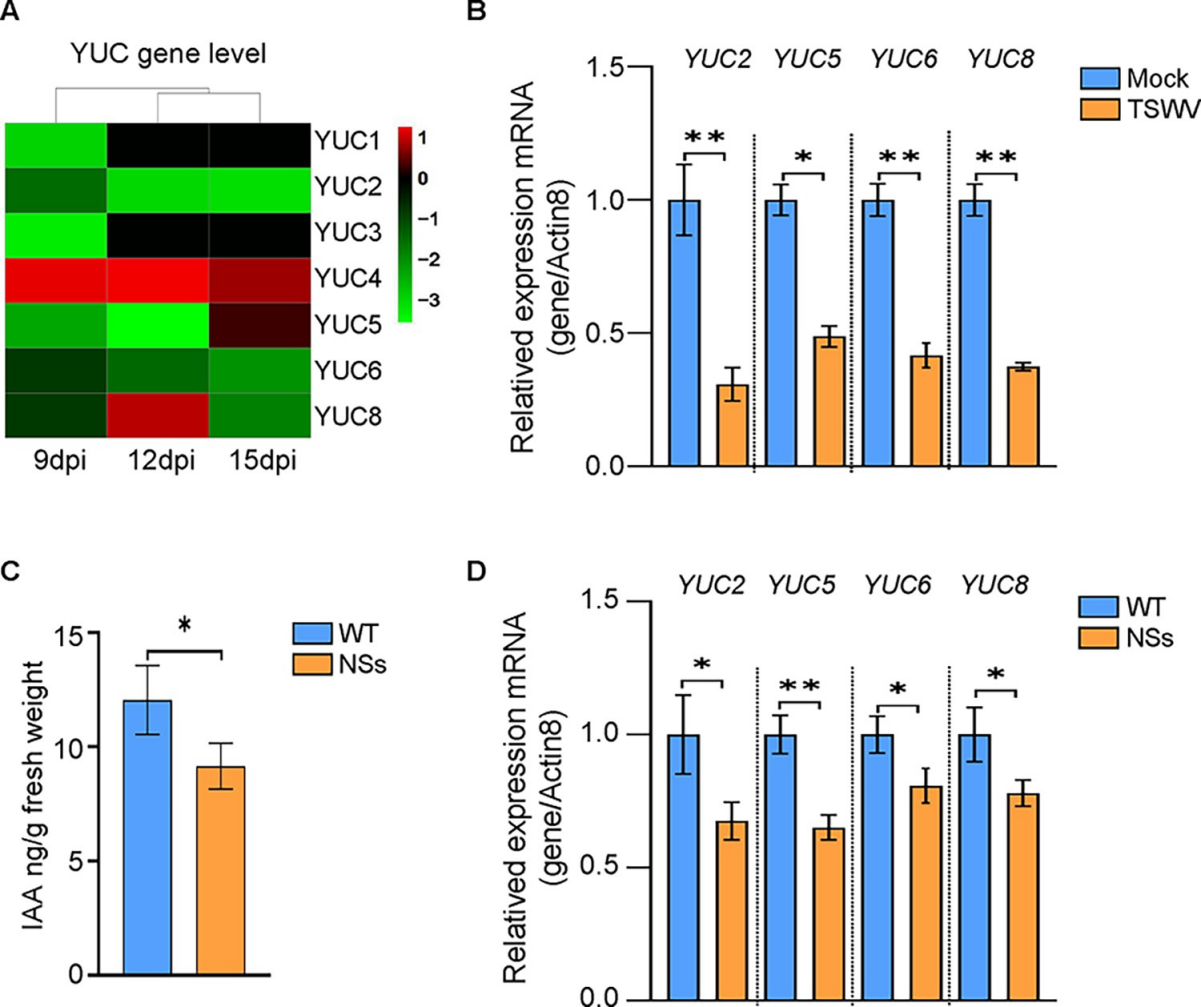
NSs interferes with the auxin synthesis pathway. (A) Analysis of published transcriptome data showed that the majority of auxin biosynthesis genes are downregulated in *Arabidopsis* plants infected with TSWV at 9, 12 and 15 dpi [73]. Gene expression up- or down-regulated are presented in red and green, respectively. (B) RT-qPCR verification of auxin biosynthesis genes in WT and TSWV infected *Arabidopsis* plants at 9 dpi. n  =  3 biologically independent samples. (C) Amount of indole-3-acetic acid (IAA) in WT and NSs-transgenic *Arabidopsis* plants. n  =  3 biologically independent samples. (D) Relative expression levels of auxin biosynthesis genes in WT and NSs transgenic plants. n  =  3 biologically independent samples. Data are mean ± s.e.m.*P < 0.05, **P < 0.01. All experiments were repeated at least three times with similar results.

The previous research found that TSWV infection can suppress the accumulation of auxin levels in plant tissues, yet the underlying molecular mechanisms need further elucidation [40]. RNA-seq analysis reveal that TSWV infection can suppress the expression of several auxin biosynthesis-related genes, known as *YUCs* [41] (Fig 1A). To confirm the reliability of the RNA-seq data, we collected Mock or virus-infected *Arabidopsis* plant systemic leaves and selected *YUC2*, *YUC5*, *YUC6*, and *YUC8* for RT-qPCR validation (S1A and S1B Fig). The results showed that the expression patterns of these *YUCs* genes were consistent with the RNA-seq data (Fig 1B). The previous research has revealed that NSs can interfere with multiple hormones signaling pathways, thereby affecting plant immunity. We sought to determine whether NSs could also interfere with the auxin synthesis pathway. To this end, we generated transgenic *Arabidopsis* plants that stably express NSs (S2A and S2B Fig). Interestingly, the content of auxin in these genetically modified plants had indeed decreased (Fig 1C). Given the interference of TSWV NSs with the auxin synthesis pathway, we explored whether exogenous application of auxin can trigger resistance against TSWV. The results indicate that exogenous application of auxin indeed enhances the resistance of *N*. *benthamiana* and *Arabidopsis* plants to TSWV (S3 Fig). In addition, the expression of *YUCs* genes was significantly downregulated in NSs transgenic plants (Fig 1D). We also measured the transcription levels of auxin-responsive genes *IAA3*, *SAUR22*, and *IAA29*, and found that they are downregulated in NSs transgenic plants (S4 Fig). The results indicate that TSWV NSs interfere with both auxin biosynthesis and signaling pathways.

### NSs interacts with TCP17 in vivo or vitro

**Fig 2:**
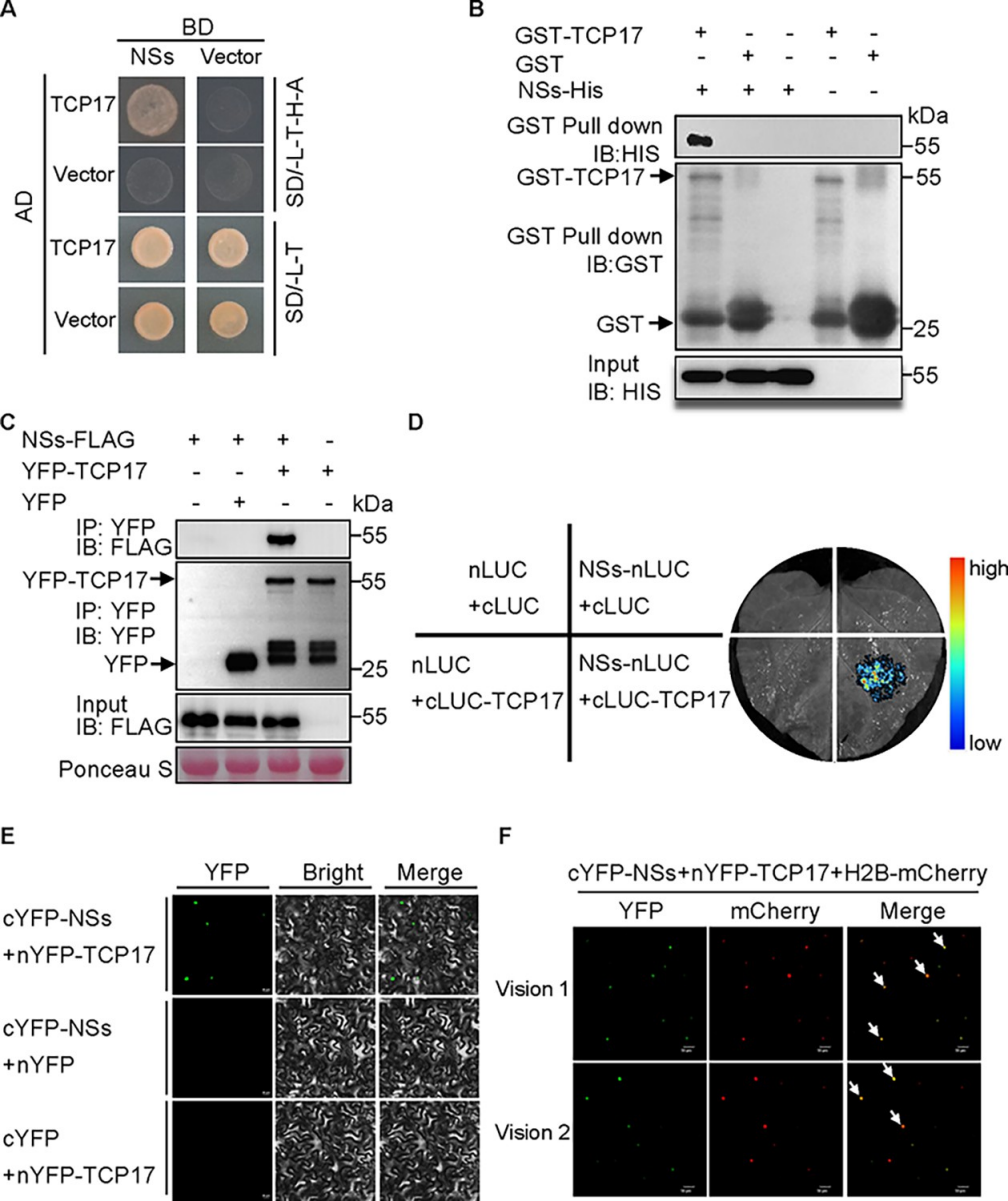
TSWV NSs protein interacts with TCP17 in vitro or vivo. (A) Y2H assays illustrating the interaction between NSs and TCP17 proteins. AD, activation domain; BD, DNA-binding domain. The yeast co-transformed with BD- and AD-derivative constructs was plated on SD/-L-T-H-A and SD/-L-T. (B) GST pull-down analysis of the interaction of NSs with TCP17. (C) Co-IP assays confirm that NSs interacts with TCP17 in *N*. *benthamiana* leaves. Total proteins were extracted and immunoprecipitated by anti-YFP magnetic beads. The coimmunoprecipitated proteins were detected by anti-FLAG antibody. (D) SLC analysis of the interaction between NSs and TCP17 in planta. cLUC- TCP17 or cLUC control vector were co-expressed with nLUC-NSs or nLUC control vector in *N*. *benthamiana* plant leaves. Luciferase activity was detected at 48 hours post-inoculation (hpi). (E) BiFC analysis of the interaction between NSs and TCP17 in planta. nYFP- TCP17 or nYFP control vector was co-expressed with cYFP-NSs or cYFP control vector in *N*. *benthamiana* plant leaves. The reconstituted YFP fluorescence signals were examined by confocal microscopy and photographed at 48 hpi. Scale bar, 20 μm. (F) NSs interacts with TCP17 in the nucleus assayed by BiFC. cYFP-NSs and nYFP- TCP17 were co-expressed with H2B-mCherry, a nuclear marker, in *N*. *benthamiana* plant leaves. The white arrow indicates the co-localization of NSs, TCP17 and H2B in the nucleus. Scale bar, 50 μm.

To elucidate the mechanism by which NSs suppresses auxin synthesis, we screened an *Arabidopsis* Y2H library using NSs as bait. This screen identified TCP17, an auxin synthesis-related transcription factor, as an interactor with NSs (Fig 2A). The interaction between TCP17 and NSs was further confirmed by in vitro glutathione S-transferase (GST) pull-down (Fig 2B). Co-immunoprecipitation (Co-IP), split-luciferase complementation (SLC) and bimolecular fluorescence complementation (BiFC) assay demonstrated that NSs indeed interacts with TCP17 in planta (Fig 2C–2E) and specifically with the full length of TCP17 (S5 Fig). The interaction between NSs and TCP17 occurred mainly in the nucleus (Fig 2F). The homologs TCP13 & 5 and more distantly TCP8 & TCP22 that are related to TCP17 [42], cannot physically interact with NSs (S6 Fig). These results demonstrate that NSs specifically binds to TCP17.

### TCP17 positively regulates the expression of genes involved in auxin biosynthesis and signaling response

**Fig 3:**
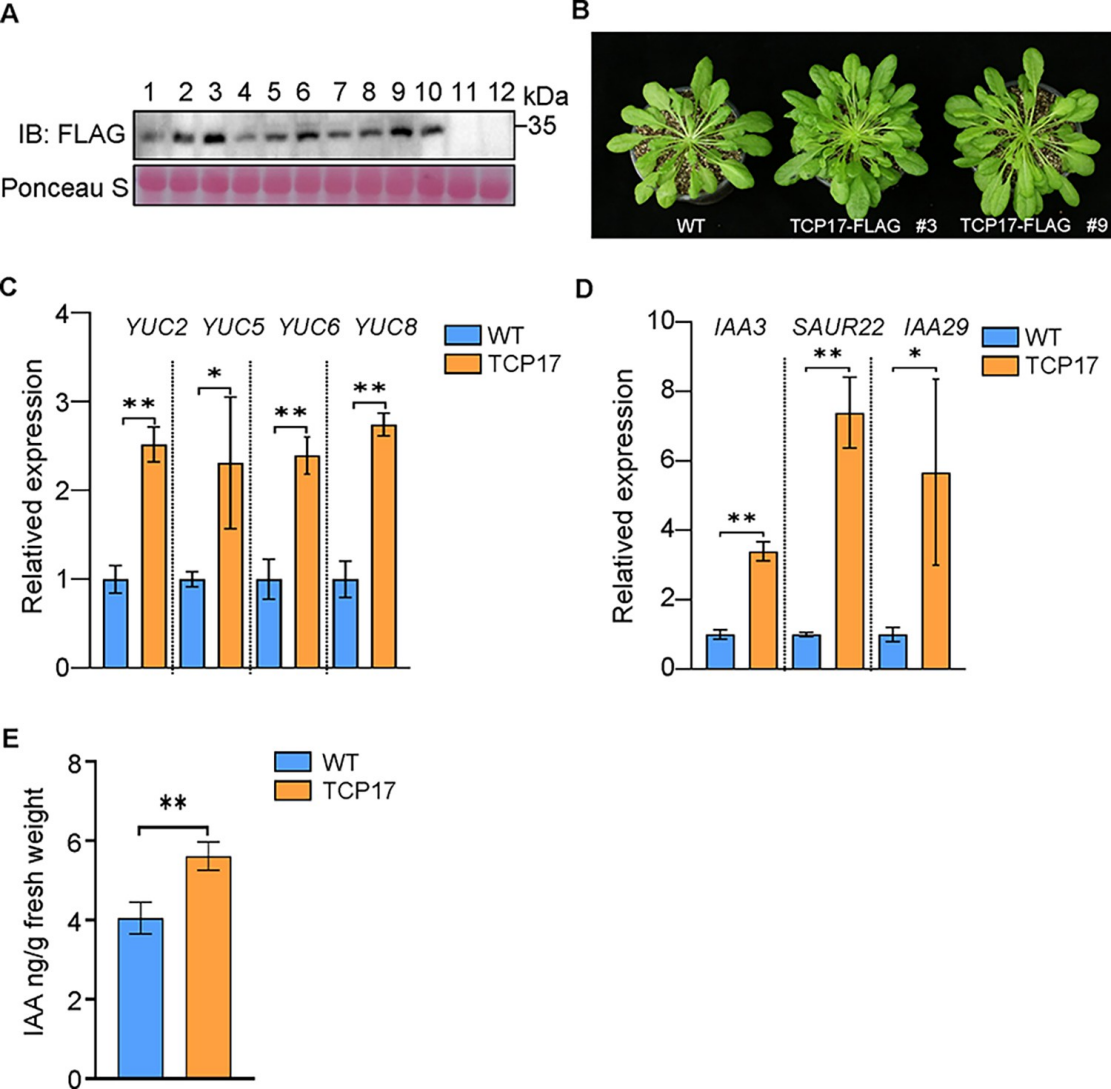
TCP17 positively regulates auxin synthesis and signaling pathways. (A) Generation of transgenic *Arabidopsis* overexpressing TCP17. Accumulation of TCP17 in 10 independent TCP17-transgenic lines was analyzed by Western blot using FLAG specific antibodies (Top). Ponceau S staining was used to estimate sample loading (down). (B) The phenotype of transgenic *Arabidopsis* overexpressing TCP17- FLAG photographed at 8-week-old stage was shown in the bottom. Lower left is the WT plant; lower middle and right are the TCP17 transgenic *Arabidopsis* lines #3 and #9. (C) Relative expression levels of auxin biosynthesis genes in WT and TCP17 transgenic *Arabidopsis* plants. Data are presented as mean values ± s.e.m.; n  =  3 biologically independent samples. (D) Relative expression levels of auxin response genes in WT and TCP17 transgenic *Arabidopsis* plants. Data are presented as mean values ± s.e.m.; n  =  3 biologically independent samples. (E) Amount of indole-3-acetic acid (IAA) in WT and TCP17 transgenic *Arabidopsis* plants. n  =  3 biologically independent samples. *P < 0.05, **P < 0.01. All experiments were repeated at least three times with similar results.

To further investigate the role of TCP17 in the auxin pathway, we generated *Arabidopsis* lines overexpressing TCP17-FLAG (Fig 3A and 3B). The immunoprecipitation experiment confirmed the stable expression of TCP17-FLAG (Fig 3A). RT-qPCR experiments indicates that TCP17 significantly upregulates the expression of several auxin biosynthesis genes (Fig 3C). The expression of auxin-responsive genes was also significantly up-regulated in TCP17 transgenic plants (Fig 3D). In addition, the accumulation level of auxin was higher in these TCP17 transgenic plants (Fig 3E). During auxin signaling, the auxin receptor TIR1 binds to and target transcriptional repressors, such as Aux/IAA protein, for degradation by the 26S proteasome upon hormone perception [8,15]. We also detected *YUCs* and auxin-responsive genes in the *tcp17* mutant, and the expression levels of *YUC5*, *YUC8*, and *IAA3* were downregulated (S7A–S7C Fig). These results indicate that TCP17 can promote the accumulation of auxin and activates the auxin signaling pathway.

### TCP17 enhances *Arabidopsis* defense against TSWV infection

**Fig 4:**
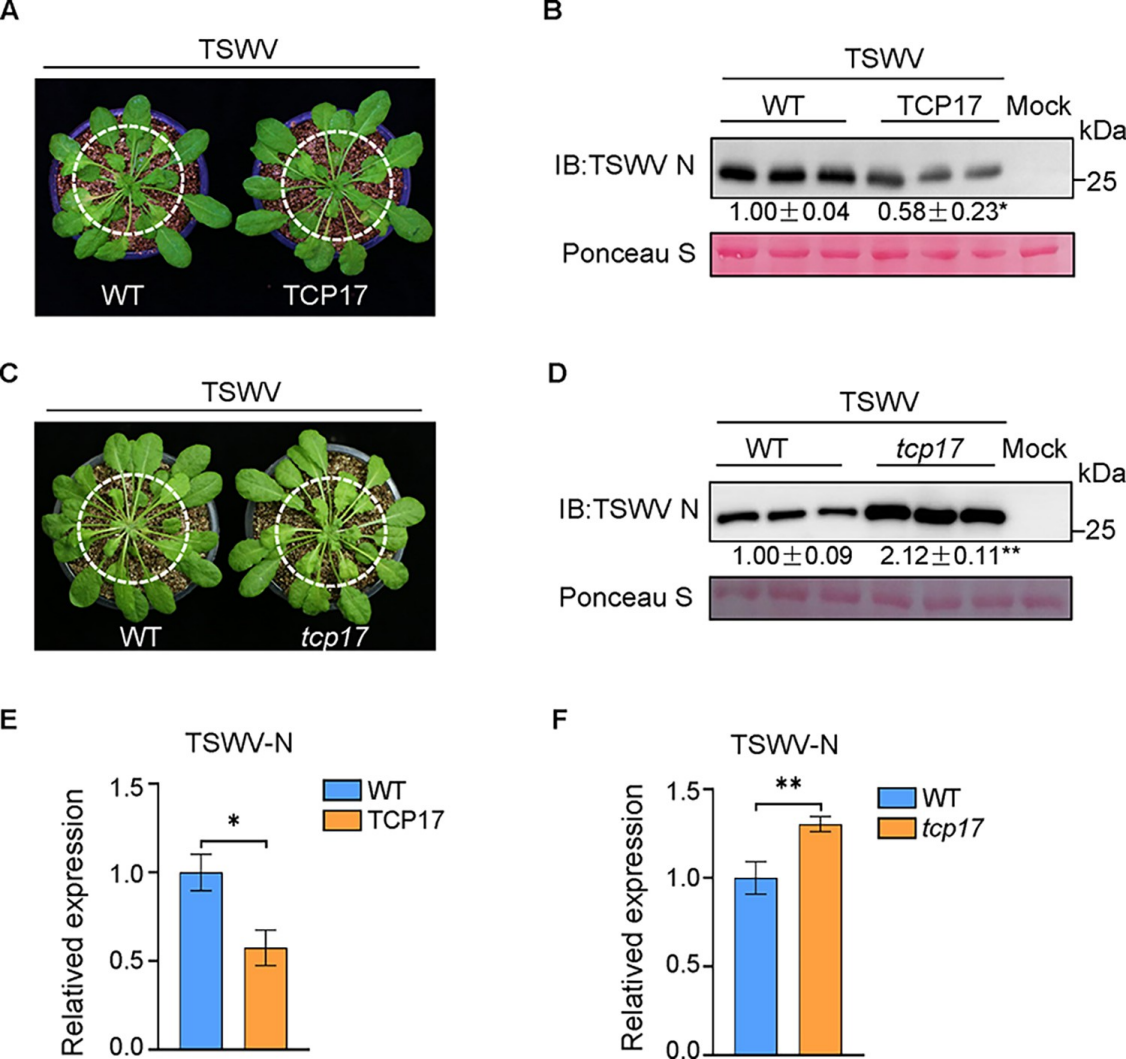
TCP17 positively modulates *Arabidopsis* resistance to TSWV. (A and C) The symptoms on TSWV-infected WT, transgenic, and mutant plants. The phenotypes were observed and photos taken at 15 dpi. (B and D) The accumulation of TSWV N protein in TSWV-infected plants determined by Western blotting. Total protein extracts were separated by SDS-PAGE and analyzed by an immunoblotting approach using an anti-N antibody. Ponceau S staining was used to estimate sample loading. (E and F) RT-qPCR results showing the relative expression levels of TSWV N in TSWV-infected transgenic and mutant plants compared with TSWV-infected control plants. *Actin8* was used as the internal reference gene to normalize the relative expression. Data are mean ± s.e.m.*P < 0.05, **P < 0.01. All experiments were repeated at least three times with similar results.

Our previous research indicates that the auxin signaling pathway plays a crucial role in resisting TSWV infection. As a key regulatory factor in the auxin signaling pathway, TCP17 is investigated for its potential role in defending against TSWV infection. In TCP17-overexpressing *Arabidopsis* plants in which auxin pathways was activated, we observed milder symptoms compared to the WT plants (Fig 4A). Immunoprecipitation assays using TSWV N antibodies revealed lower levels of TSWV in the transgenic line (Fig 4B). The RT-qPCR data also demonstrated similar results (Fig 4E). Thus, *Arabidopsis* overexpressing TCP17 was less susceptible to viral infection.

To further confirm the role of TCP17 in the virus infection process, *tcp17* mutants and WT plants were also inoculated with TSWV. After challenge with TSWV, the mutant plants had more severe symptoms than the controls (Fig 4C). The mutants also had a greater accumulation of TSWV N protein and higher concentration of TSWV genomic RNAs (Fig 4D and 4F). Overall, these results suggest a critical role for TCP17 in enhancing host defense against TSWV.

### NSs interferes with auxin biosynthesis by suppressing the transcriptional activation activity of TCP17

**Fig 5:**
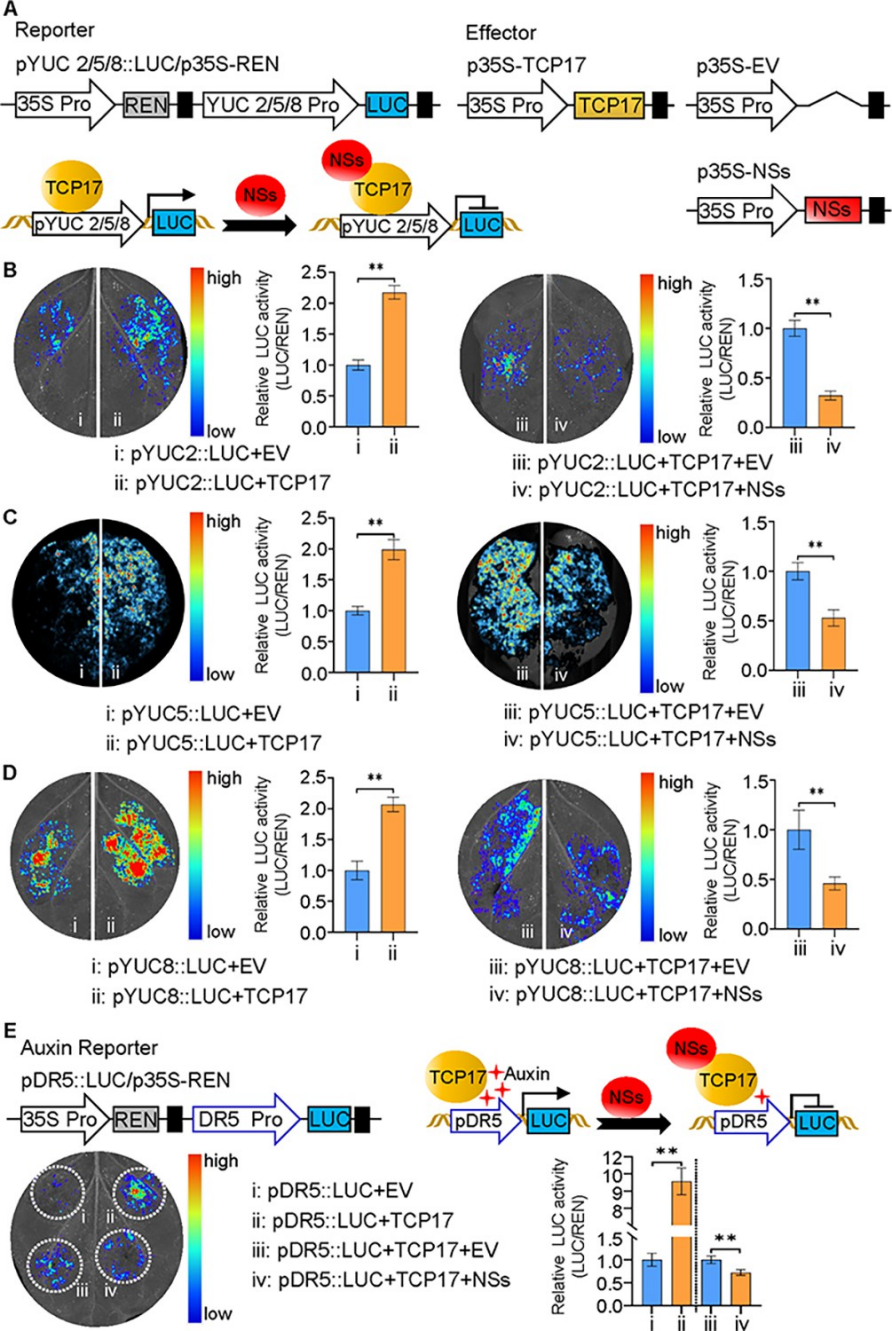
NSs suppresses the transcriptional activation of TCP17 to attenuate auxin synthesis and signaling pathways. (A) Schematic diagrams of the effectors and reporters used in the dual-LUC experiments. (B-D) The relative LUC activities were measured in *N*. *benthamiana* cells, using the combinations shown in A. The empty BD effector was used as a negative control. The LUC/REN ratio represents the relative LUC activity. The concentration of agrobacterium individually carrying those constructs were used at OD_600_ =  1.0. The luciferase activity was assayed at 48 hpi. The luciferase activity in the treated leaves was quantified and shown in the right. Data are presented as mean values ± s.e.m.; n  =  3 biologically independent samples. **P < 0.01. (E) Transient overexpression of TCP17 transcription factor activated the expression of luciferase (LUC) driven by the DR5 promoter, and addition of NSs into TCP17 reduced the expression of LUC. The luciferase activity in the treated leaves was quantified and shown in the right. Data are presented as mean values ± s.e.m.; n  =  3 biologically independent samples. **P < 0.01.

As a transcriptional activation factor, TCP17 positively regulates the auxin synthesis pathway [31]. NSs, which interacts directly with TCP17, may inhibit auxin synthesis by targeting the transcriptional activation of TCP17. To verify this hypothesis, we conducted dual-luciferase transient transcriptional activity assays (Fig 5A). TCP17 strongly activated the luciferase (LUC) reporter gene driven by the promoter of *YUC2*, *YUC5* and *YUC8* (Fig 5B–5D), but not a mutation of *YUC5* promoter in the TCP17 binding site and the promoters of *ACTIN2* and *ACTIN5* (S8 Fig). Interestingly, both the *SlYUC5* and *CaYUC5* promoters contain binding sites for TCP17 and can be activated for expression by SlTCP17 and CaTCP17, respectively (S9 Fig). Notably, the addition of NSs to the luciferase reporter system significantly inhibited the expression of the reporter gene activated by TCP17 compared to the control (Fig 5B–5D), and this inhibition was stronger than that observed before TCP17 activation alone (S10 Fig). This may be due to the fact that NSs strongly repress the activation of the YUC promoter by TCP17, including plant endogenous TCP17. *DR5* has been reported as an synthetic auxin-responsive reporter gene [43]. Exogenous application of auxin can activate the expression of the luciferase reporter gene driven by the *DR5* promoter (S11A Fig). At the same time, YUC2, YUC5, and YUC8 can also activate the expression of this reporter gene (S11B Fig). Overexpression of TCP17 enhances the expression of the *DR5* reporter gene, while the addition of NSs suppresses luciferase expression (Fig 5E). These results indicate that NSs disrupts the transcriptional activation activity of TCP17 and suppress the expression of genes involved in auxin synthesis.

### NSs attenuates auxin biosynthesis by interfering with TCP17 dimerization

**Fig 6:**
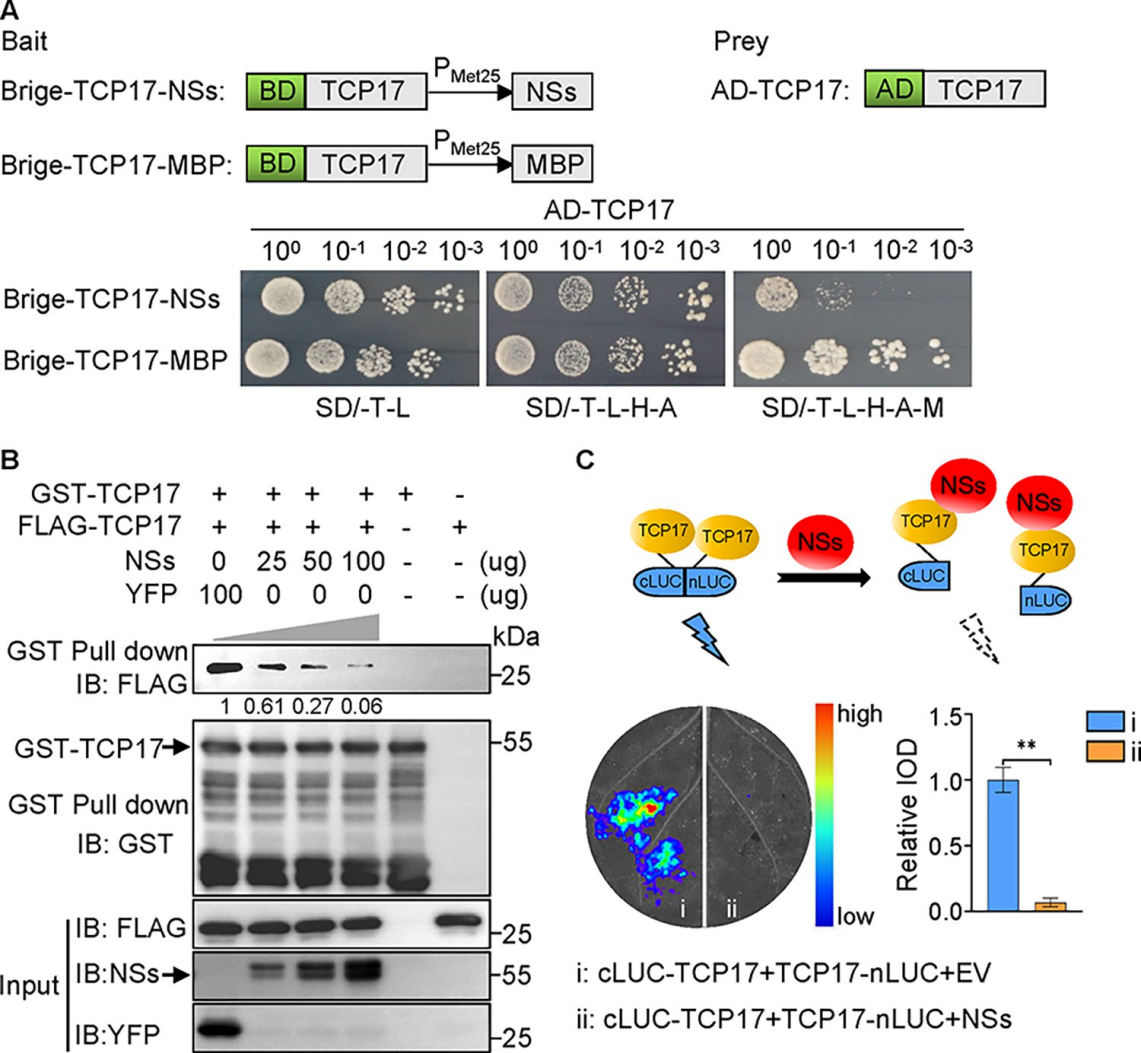
NSs interferes with TCP17 dimerization. (A) Y3H assay results showing the effects of the NSs on TCP17 dimerization. (Upper) Schematic diagrams of the bait and the prey constructs used in Y3H assays. Yeast cells were co-transformed with pGAD-TCP17 and pBridge-TCP17+MBP, or pGAD-TCP17 and pBridge-TCP17+NSs. (Lower) The co-transformed yeast cells were grown on the SD/-T-L, SD/-T-L-H-A, or SD/-T-L-H-A-M (lacking Trp, Leu, His, Ade, and Met) plates, respectively, for 5 d. (B) GST pull-down assay results showing the effect of NSs on TCP17 dimerization. Fixed amount of GST-TCP17 and FLAG-TCP17 was incubated with increasing amounts of purified NSs or purified YFP (control). Proteins in the samples were then pulled down using glutathione-sepharose beads followed by western blot assays with GST-, Flag-, NSs- and YFP-specific antibodies. (C) SLC assays demonstrating the influence of NSs on TCP17 dimerization. The schematic diagram of the experiments is shown in the upper left. Co-expression of TCP17-nLUC and cLUC-TCP17 produce luciferase activity (indicated by a thunder). TCP17-nLUC and cLUC-TCP17 were used to co-express with NSs or pCambia2300S empty vector (EV) in *N*. *benthamiana* plant leaves (lower left). The concentration of agrobacterium individually carrying those constructs were used at OD_600_ =  1.0. The luciferase activity was assayed at 48 hpi. The luciferase activity (Integrated Optical Density, IOD) in the treated leaves was quantified and shown in the lower right. Data are presented as mean values ± s.e.m.; n  =  3 biologically independent samples. **P < 0.01.

Transcription factors exerts their transcriptional activation function through self-dimerization [20]. To further explore whether NSs affected the dimerization of TCP17, we validated this using yeast three-hybrid (Y3H) assays. The results indicate that the self-interaction of TCP17 is significantly inhibited in the presence of NSs compared to the control MBP protein (Fig 6A). The GST pull-down assay result also showed that when the amount of NSs was increased, the amount of FLAG-TCP17 pulled down by GST-TCP17 was decreased, but the addition of YFP had no clear effect on self-interaction of TCP17 (Fig 6B). In addition, SLC assays confirmed that the co-expression of NSs reduced the self-interaction of TCP17 (Fig 6C). Besides interfering with the self-dimerization, does NSs have any impact on the transcription binding of TCP17? Previous research has found that class-I and class-II TCPs preferentially bind to the DNA motifs GGNCCCAC and GTGGNCCC, respectively. We selected potential transcription binding sites (657bp to 599 bp upstream of the transcription starting codon ATG) on the *YUC5* promoter for validation. The EMSA experimental results showed that the intensity of the shifted bands weakened with the addition of increasing amounts of unlabeled wild-type probes, but mutated probes did not lead to this reduction, indicated that TCP17 can specifically bind to the transcription binding site of the *YUC5* promoter (S12A Fig). However, the addition of NSs did not directly affect the activity of TCP17 binding to DNA fragments (S12B Fig). These results indicated that NSs inhibits the transcriptional activation of TCP17 by interfering with its dimerization.

To demonstrate the significance of the identified interaction on virus biology, we used AlphaFold3 to predict the interaction interface and sites of NSs with TCP17, and mutated the corresponding residues. (S13A and S13B Fig). The Y2H results show that the NSs mutant is unable to interact with TCP17 and the reporter system of the infectious clone containing the NSs mutant exhibits weaker expression compared to that of the infectious clone with the wild-type NSs. (S13B–S13D Fig). Therefore, the interaction between NSs and TCP17 plays a crucial role in maintaining the accumulation of TSWV.

**Fig 7:**
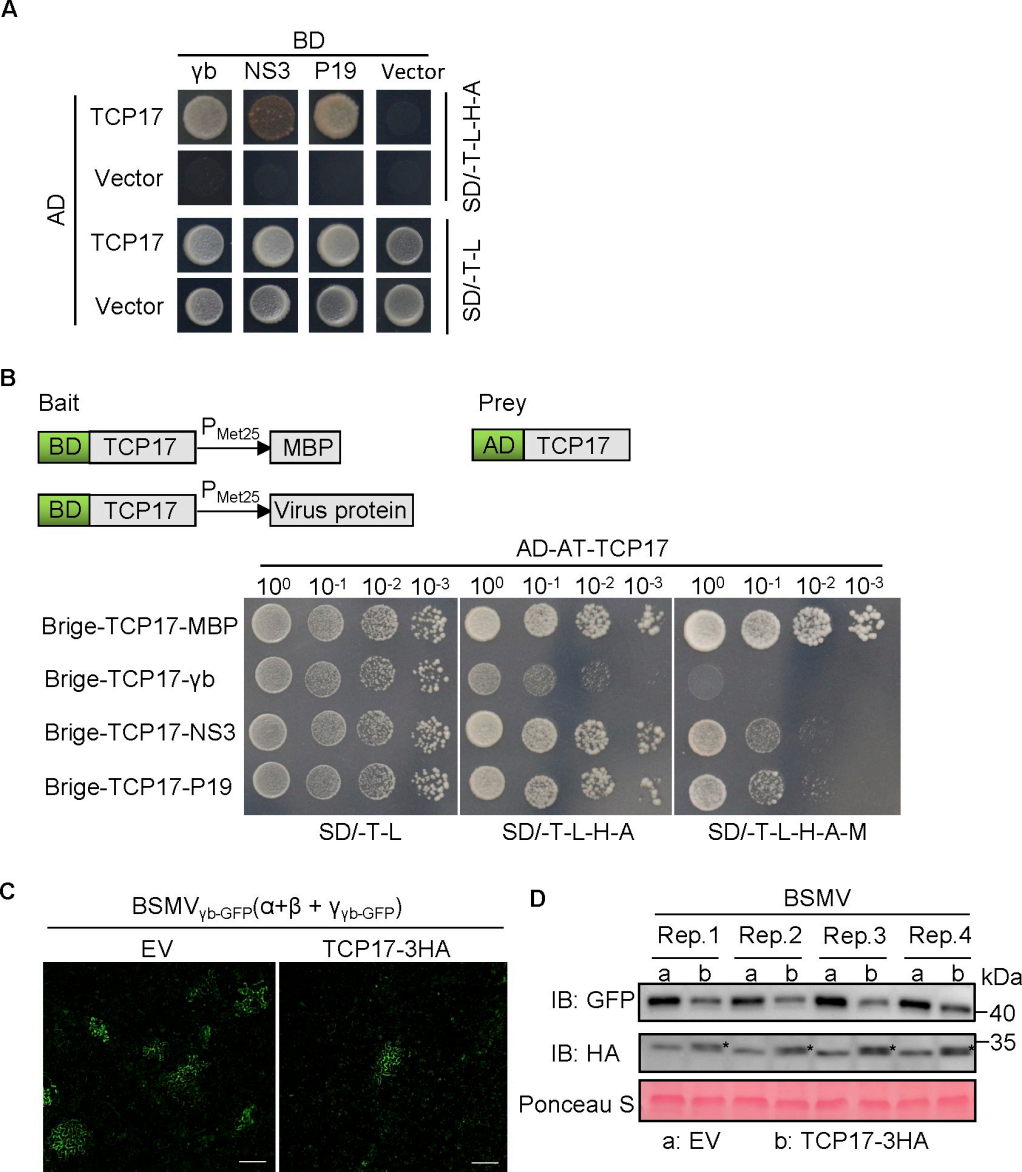
Inhibiting dimerization of TCP17 by viral suppressors of RNA silencing (VSR) is conserved in plant viruses. (A) Y2H assays illustrating the interaction between several VSRs and TCP17 proteins. Viral proteins were fused with BD while TCP17 was cloned into AD yeast vectors. All transformants were selected on SD-L-T-H-A plates at 30°C and photographed after 5 days. (B) Y3H assay results showing the effects of the viral proteins on TCP17 dimerization. (Upper) Schematic diagrams of the bait and the prey constructs used in Y3H assays. Yeast cells were co-transformed with pGAD-TCP17 and pBridge-TCP17+MBP or pBridge-TCP17+γb, pBridge-TCP17+NS3 and Bridge-TCP17+P19. (Lower) The co-transformed yeast cells were grown on the SD/-T-L, SD/-T-L-H-A, or SD/-T-L-H-A-M (lacking Trp, Leu, His, Ade, and Met) plates, respectively, for 5 d. (C) Effects of TCP17-overexpression on BSMV infection. BSMV γb-GFP was agroinfiltrated into *N*. *benthamiana* together with TCP17-3HA or EV (Empty vector). The GFP fluorescence indicates BSMV-infected cells and was photographed by confocal microscope at 60 hpi. Bars, 50 μm. (D) Western blot assay results showing the accumulation level of GFP at 60 hpi in the infiltrated leaves shown in (C), using anti-GFP antibody. The black asterisk indicates TCP17-3HA bands. Ponceau S staining was used to estimate sample loading.

To investigate whether plant virus attacking TCP17 is a conservative strategy, we performed Y2H assays to test for potential interactions between the gene silencing suppressors of several RNA viruses and TCP17.The research findings indicate that BSMV γb, RSV NS3, and TBSV P19 can interact with TCP17 (Fig 7A). We then used Y3H assay to test the abilities of different gene silencing suppressors to disrupt the self-interaction of TCP17. The results indicate that BSMV γb, RSV NS3, and TBSV P19 can all inhibit the dimerization of TCP17 (Fig 7B). To understand the role of TCP17 during BSMV infection, *N*. *benthamiana* leaves were co-infiltrated with Agrobacterium harboring a BSMV γb-GFP infectious clone and a construct expressing TCP17-3-HA or an empty vector (EV). By 2.5 days post-inoculation (dpi), the accumulation of BSMV was significantly lower in the regions expressing TCP17-3-HA compared to the control (Fig 7C and 7D). Thus, overexpression of TCP17 in *N*. *benthamiana* also enhanced resistance to BSMV.

## Discussion

In the enduring struggle between host plants and pathogenic microorganisms, a sophisticated set of co-evolved arms race mechanisms has emerged, which encompasses plant defense and pathogen counter-defense strategies [44–46]. Phytohormones represent a prevalent and effective class of plant defense mechanisms against pathogenic invaders, with auxins, jasmonic acid, and salicylic acid being key players in the plant’s arsenal against pathogen infection [23,47–50]. Conversely, pathogens have evolved multifunctional proteins to counteract these phytohormone-based defense responses [49–52]. However, the precise mechanisms by which manipulate host hormone levels to facilitate infection remain largely elusive.

**Fig 8:**
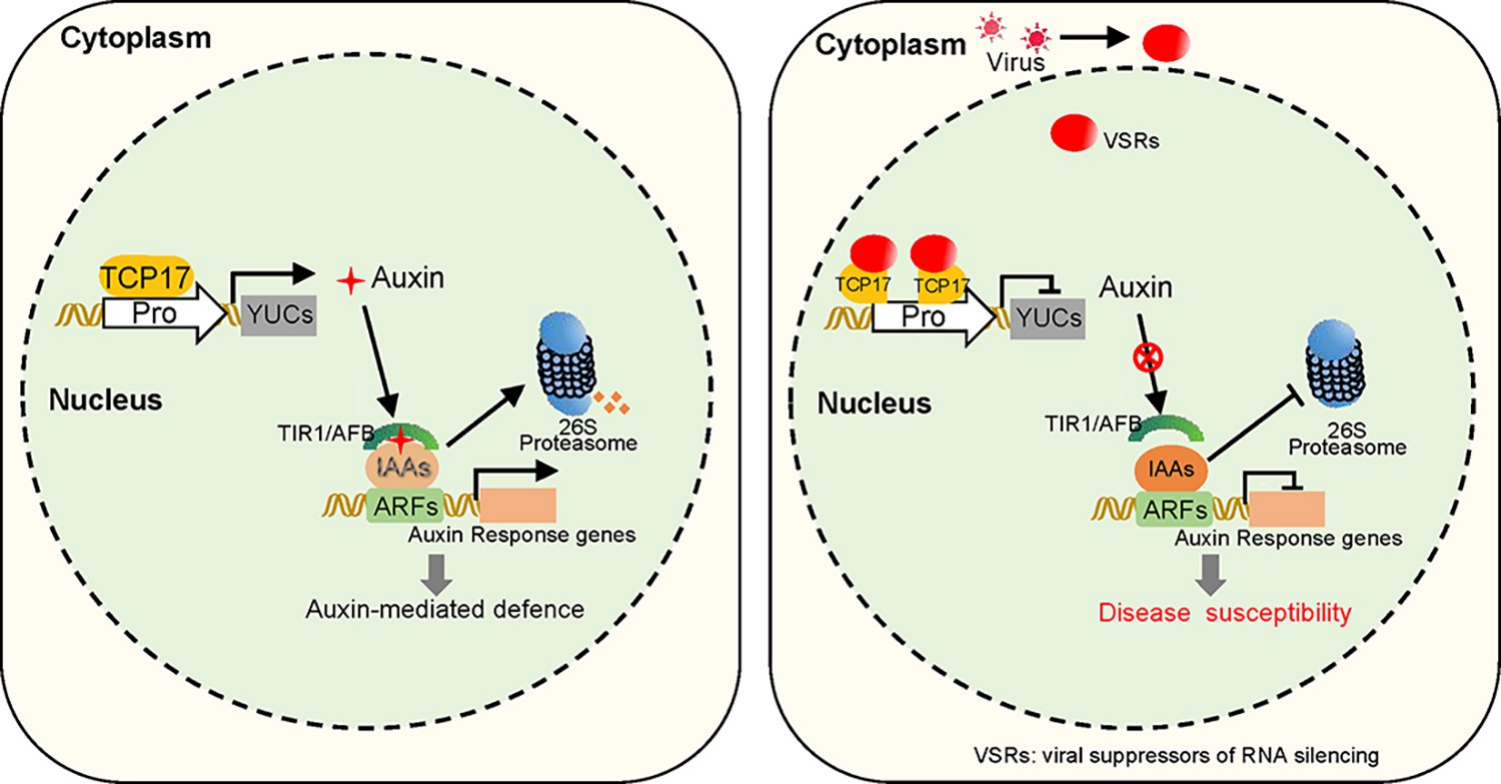
Model of VSRs suppressing host immunity by interfering with dimerization of TCP17. A model depicting how plant viruses suppress immune responses by disrupting auxin biosynthesis. On the left, the auxin pathway is crucial for defense against TSWV. On the right, virus-encoded suppressors of RNA silencing (VSRs) from various viruses can inhibit plant defense responses by targeting TCP17.

This study elucidates a novel interaction between the NSs protein of TSWV and the transcription factor TCP17, which is pivotal in repressing the expression of YUCs, a key gene in auxin synthesis. This interaction leads to a reduction in auxin content within the host plant. We further demonstrated that the NSs disrupts the dimerization of TCP17, impairing its biological functions (Fig 8). Auxin, an endogenous molecule, directly activates auxin signaling, with the auxin receptor directly binding to auxin to degrade auxin-responsive inhibitory factors, thereby activating auxin-responsive pathways [13–15,53,54]. Overexpression of TCP17 through transgenic methods revealed an increase in auxin content and enhanced expression of auxin-responsive genes Conversely, the TCP17 knockout mutant showed partial repression of the auxin response gene expression. Partial functional redundancy between TCP genes is a common phenomenon [55,56]. There may be other TCPs that compensate some of the functions of TCP17 in *tcp17* mutant. Therefore, in contrast to the transgenic plants overexpressing TCP17, it is difficult to observe changes in the expression of auxin response genes in the *tcp17* mutant. Nonetheless, the NSs transgenic overexpression plants were still able to strongly inhibit the auxin synthesis and signaling pathways. Therefore, we speculate that in addition to TCP17, NSs may also target other unknown auxin-related factors. At the same time, the TSWV infection phenotype showed that overexpression of TCP17 significantly inhibited TSWV infection, with more severe symptoms observed in the *tcp17* knockout mutants.

Our previous research reported a decrease in auxin content and inhibition of auxin signaling response in plants following TSWV infection [40], but the underlying mechanism unclear. In this study, we primarily focused on the downregulation of auxin synthesis genes following TSWV infection and observed that both auxin synthesis genes and the content of auxin were reduced in NSs transgenic plants. Transcription factors often function by forming homo- or heterodimers [20,57], and TCP proteins are no exception, forming such complexes to perform their biological roles [25,28]. We demonstrated that TCP17 can form homodimers in vivo and in vitro, and intriguingly, NSs inhibits the transcriptional activation function of TCP17 by interfering with its dimerization. Notably, the addition of NSs very strongly inhibited TCP17 activation activity and that this inhibition was stronger than that observed before TCP17 activation alone (S10 Fig). This may be due to the fact that NSs strongly suppress the activation of the YUC promoter by TCP17, including the endogenous TCP17 of the plant.

Further research showed that multiple RNA virus silencing suppressors target TCP17, suggesting that it is a widely prevalent host factor susceptible to viral attack (Fig 8). Pathogenic microorganisms often attack phytohormone response pathways to inhibit host basal defenses and promote infection, but direct reduction of phytohormone content by pathogens is rarely reported. TCP17 is a key transcription factor that promotes the expression of auxin synthesizing genes *YUCs* [31]. Viral targeting of TCP17 results in the downregulation of *YUCs* gene expression, thereby leading to a decrease in auxin content. This loss of host auxin-responsive defenses due to pathogenic attack on TCP17 represents an efficient strategy that greatly aids further pathogen infection.

Transcription factors play important roles in plant growth, development, and defense responses. Given that a large number of transcription factors exist in plants that are related to the basal defense of the host plant [36,58]. Our research presents a paradigm for pathogenic microorganisms exploit the manipulation of transcription factors as a strategy to facilitate infection. While the majority of previous studies have concentrated on the role of phytohormone signaling in the plant innate immunity against diseases, there has been a relative scarcity of research on how pathogens directly interfere with hormone levels to undermine the host’s immune response. Our study offer a novel perspective on how viral virulence factors can target transcription factors to manipulate hormone levels, thereby suppressing host hormone signaling and immunity. It remains an intriguing question whether other pathogen effectors might target transcription factor-regulated hormone levels to antagonize host immunity.

## Materials and methods

### Plant materials and plant growth conditions

The full-length coding sequences *TCP17* amplified from *Arabidopsis* cDNA was fused with 3xHA tag. *NSs* and *TCP17*-3xHA were cloned into pCAM2300S binary vector driven by 2x35S promoter to generate the pCAM2300S-TCP17-3xHA. The TCP17 constructs were transformed into WT *Arabidopsis* (Col-0) plants using the Agrobacterium-mediated floral dip method to generate the 35S:TCP17-3xHA transgenic plants. The seeds of *Arabidopsis* mutants *tcp17* were provided by AraShare (https://www.arashare.cn). The seeds were incubated at 4°C for 7 days before sowing. *Arabidopsis* plants were grown in a growth chamber at temperatures of a 16-h (25°C)/8-h (23°C) light/dark photoperiod. *N*. *benthamiana* were grown in a growth chamber at temperature of 25°C, 60% humidity and a 16 h light/8 h dark cycle.

### Expression and purification of recombinant proteins

To obtain recombinant GST-tagged TCP17 proteins, the coding sequences of *TCP17* from *Arabidopsis* was cloned into pGEX-2TK vector as instructed (GE Healthcare Life Sciences, Pittsburgh, PA, USA). To obtain NSs-FLAG and FLAG-TCP17 recombinant proteins, the coding sequences of those genes were fused with FLAG and cloned into pET-28a vector with 6xHIS (Novagen, Darmstadt, Germany). To obtain purified NSs, YFP and TSWV-N recombinant proteins, the coding sequences of those genes were cloned into pET-28a vector with 6xHIS. These constructs were introduced into *Escherichia coli* Rosetta strain (DE3). Expression and purification of recombinant proteins as described previously (Chen et al. 2023) [40]. Briefly, Rosetta cells were grown at 20°C to OD600 of 0.6–0.8 and induced with 0.1 mM IPTG for 16 h. Cells were harvested and resuspended in 10 mL Lysis Buffer (20 mM NaH2PO4.2H2O, 10 mM imidazole, 300 mM NaCl, PH 8.0). The lysate was decomposed by ultrasound and then centrifuged at 8000 rpm for 30 min. 150 μL Glutathione Resin (Thermo Fisher) or Ni-NTA resins (Qiagen) were added to the solution and incubate at 4°C for 2 h. The resins were harvested and resuspended in 12 mL 1xPBS buffer, then poured into Chromatography Columns (Bio-Rad).

### Antibody preparation

For the detection of NSs, TSWV-N, GST, and YFP, these proteins were fused with 6xHIS tag and were expressed in *Escherichia coli* Rosetta (DE3) and then purified using Ni-NTA agarose (QIAGEN, Venla, Netherlands) as instructed [59]. Purified TSWV NSs protein was injected into mouses to produce polyclonal antibodies. Purified TSWV-N, GST, and YFP protein were injected into rabbits to produce polyclonal antibodies. The collected serum was centrifuged for 10 min at 5000 rpm and the supernatant from each sample was stored at -20°C.

### Yeast two-hybrid (Y2H) cDNA library screening and assay

Yeast two hybrid screening was performed as reported previously [40]. Briefly, the construct pGBKT7 expressing NSs was transformed into Y2H gold yeast cells and grown at 30°C for 24 h on the SD/-Trp plates. The cells were pelleted through centrifugation at 3,000 rpm for 5 min and resuspended in 5 mL of fresh SD/-Trp medium. One milliliter of *Arabidopsis* thaliana cDNA yeast library was mixed with 5 mL Y2H Gold yeast carrying pGBKT7-NSs. The mating cultures were then grown with 50 μg/mL kanamycin, 0.003% adenine and 45 mL 2xYPD medium at 30°C for 24 h. The cells were collected by centrifugation at 3,000 rpm during 10 min and resuspended in 10 ml of 0.5× YPD liquid medium. Ten milliliters of mating culture (200 μl per plate) were plated on total of 50 plates of 150 mm diameter with SD/-Trp-Leu-His medium and then incubated at 30°C for 5–7 days. Plasmid DNA from positive prey clones was purified by TIANprep Yeast Plasmid DNA Kit (DP112, TIANGEN BIOTECH, China). Identities of the NSs-interacts were confirmed through the NCBI database (https://www.ncbi.nlm.nih.gov).

For Y2H assay, the coding sequences of full-length *NSs*, *γb*, *NS3*, and *P19* were fused to the GAL4 binding domain and cloned in the pGBKT7 vector as the bait, and the TCP17 from *Arabidopsis* were fused to the GAL4 activation domain and cloned in the pGADT7 vector the prey. The bait and prey constructs were co-transformed into yeast cells and grown on synthetic defined (SD) yeast Leucine and Tryptophane double dropout medium (SD/-L-T) at 30°C for 3 d. Weak and strong interactions were examined by plating yeast transformants on the SD/-Trp-Leu-His-Ade (SD/-T-L-H-A) plates at 30°C for 6 d.

### Co-immunoprecipitation (Co-IP) assay

The full-length coding sequences of *NSs* and *TCP17* were cloned into the vector pCAM2300 with a Flag tag or YFP tag, and the resulting constructs were transformed into Agrobacterium tumefaciens GV3101 [60,61]. The Agrobacterium tumefaciens were transiently co-expressed in the leaves of 3- to 4-week-old *N*. *benthamiana* plants 48 hours after an OD600 of 0.5 was reached. Total proteins were extracted from leaves using extraction buffer. The extracted proteins were incubated for 1.5 hours at 4°C using Flag-Trap beads (Sigma) and then washed three times with Co-IP buffer (0.1% Triton X-100, 1 mM DTT, 10% glycerol, 25 mM Tris-HCl, pH 7.5, 150 mM NaCl, 1 mM EDTA). The precipitated proteins were eluted with the SDS loading buffer. The immunoprecipitants were then denatured by the addition of 5× protein loading buffer containing β-mercaptoethanol and separated on a 10% SDS–PAGE gel. Anti-YFP (AlpalifeBio), anti-Flag (Sigma) or anti-rabbit (Sigma) antibodies were used for immunoblot analysis. The SuperSignal West Pico PLUS Chemiluminescent Substrate (Thermo Scientific) was used for detection. All of the experiments were repeated three times.

### Pull-down assay

The full-length coding sequences of *NSs* and *TCP17* were cloned into the vectors pGEX-2TK and pET28a, respectively. The plasmids pGEX-2TK, pET28a, pGEX-2TK-NSs, and pET28a-TCP17 were expressed in E.coli strain Rosetta. The transformed Rosetta cells were grown separately in Luria-Bertani broth at 20°C to an OD of 0.6–0.8, and induced with 0.1 mM IPTG for 16 h. For the pull-down assay, the bacteria were collected by centrifugation at 4,500 g for 20 minutes and suspended in lysis buffer. The suspension was then sonicated and centrifuged at 18,000 g for 1 hour. The supernatant containing GST-TCP17 was incubated with 40 μL GST magnetic beads (Thermo Scientific) for 1 hour at 4°C. After washing the beads five times with PBS, the bound proteins were eluted by boiling in 5× protein loading buffer and then subjected to immuno-blotting analysis. The signals were visualized as described previously [62,63].

### BiFC assay

The bimolecular fluorescence complementation (BiFC) methods were described in the previously mentioned study [64,65]. For the BiFC assays, the full-length coding sequences of NSs and TCP17 were cloned into the binary vectors nYFP or cYFP [66], respectively. These constructs were then transformed into strain GV3101 and transiently co-expressed with various combinations in the leaves of *N*. *benthamiana*. The YFP fluorescence signal for each combination was detected after 48 hours of infiltration using an inverted confocal microscope (OLYMPUS IX81).

### Split-luciferase (SLC) assay

The SLC assays were performed as described [67,68]. For the SLC assays, the full-length coding sequences of *NSs* and *TCP17* were cloned into the vectors pCAM1300-nLUC and pCAM1300-cLUC [69], under the control of the 35S promoter. These plasmids were transformed into Agrobacteria GV3101. Equal volumes of A. tumefaciens harboring NSs-nLuc, cLuc-NSs, TCP17-nLuc, or cLuc-TCP17 were mixed at OD_600_ = 0.5. The mixed suspension was infiltrated into *N*. *benthamiana* leaves. After 2 days of incubation, the leaves were sprayed with 1 mM D-luciferin (Yesen) dissolved in 0.01% (v/v) Triton X-100 and kept in the dark for 10 minutes before signal detection with a Tanon-5200 imaging system. The luciferase activity was analyzed using ImageJ software. Each split-LUC assay was performed with at least 3 leaves.

### Total RNA extraction, RT-PCR, and RT-qPCR

Total RNA was isolated from the *Arabidopsis* leaves using FreeZol Reagent (catalog no. R711-01, Vazyme Biotech, Nanjing, China). First-strand cDNA was synthesized using a III 1st Strand cDNA Synthesis Kit (catalog no. R312-01, Vazyme Biotech, Nanjing, China). PCR was performed using gene specific primers. The resulting PCR products were visualized in 1.5% agarose gels through electrophoresis. Quantitative RT-PCR was performed with Bio-Rad CFX96 real-time PCR system using Green Master Mix (catalog no. 11201–11203, Yeasen Biotech, Shanghai, China) with cycle: 95°C for 5 min, 40 cycles of 95°C for 10 secs, 60°C for 30 secs. The expression levels of actin gene were used as internal control. The RT-qPCR data were analyzed using the 2^-ΔΔCt^ method [70]. The primers used for RT-qPCR are listed S1 Table. All RT-qPCR assays were performed using three bioreplicate samples.

### Virus inoculation

For TSWV inoculation, the source of Agrobacterium culture carrying the full-length tomato spotted wilt virus (TSWV) infectious clone L(+)opt, M(–)opt, and SR(+)eGFP was reported previously [71]. For BSMV inoculation, the OD600 of Agrobacterium containing each RNA segment is 0.3. The source of TSWV lettuce isolate was described in a recent report and maintained in *N*. *benthamiana* plants [70]. For TSWV infection, the fresh *N*. *benthamiana* tissues infected by TSWV were grounded in 1xPBS buffer (137 mM NaCl, 2.7 mM KCl, 10mM Na_2_HPO_4_, 2 mM KH_2_PO_4_. pH 7.4) and rub-inoculated onto plant leaves of 6-week-old *Arabidopsis* dusted with silicon carbide. The TSWV-infected and mock-inoculated plants were kept in a growth chamber at temperatures of a 16-h (25°C)/8-h (23°C) light/dark photoperiod.

### Electrophoretic mobility shift assay (EMSA)

The promoter fragments from *YUC5* containing GGNCCC motif was synthesized (Sangon Biotech, Shanghai, China) and labeled with Alexa Fluor 680 at their 5′ end. 6× His-TCP17 was produced in E. coli strain BM Rosetta (DE3) and purified with Ni-NTA resins (Qiagen). Briefly, Alexa Fluor 680-labeled probes were incubated with recombinant 6× His-TCP17 in binding buffer (10 mM Tris, 10 mM H3BO3. pH 8.4) for 20 min at 28°C, and the free and bound probes were separated on a 0.8% agarose gels. The shifted signals were detected using Imaging System (Odyssey DLx).

### Hormone treatments

*Arabidopsis* or *N*. *benthamiana* plant leaves were pretreated with DMSO, 50 μM indole-3-acetic acid (IAA; macklin, Shanghai, China). After 24 h, *Arabidopsis* or *N*. *benthamiana* plant leaf samples were collected and analyzed using RT-qPCR or western-blot assay, Three biologically independent replicates of each set of experiments [72,73].

### Yeast Three-Hybrid (Y3H) assay

For Y3H assays, the CDS sequence of *NSs*, *γb*, *NS3*, *P19* and *MBP* was ligated to the pBridge vector (Clontech, CA, USA), respectively, and then the CDS sequence of *TCP17* was ligated into the pBridge vector, constructing the recombination plasmids pBridge-TCP17-NSs, pBridge-TCP17-γb, pBridge-TCP17-NS3, pBridge-TCP17-P19 and pBridge-TCP17-MBP, respectively. The full length of *TCP17* was ligated to pGADT7 vector. Transformed AD-TCP17 separately with BD-TCP17-NSs, BD-TCP17-γb, BD-TCP17-NS3, BD-TCP17-P19, and BD-TCP17-MPB into the Y2H-gold yeast strain, and incubated at 30°C on SD/-Trp-Leu medium for 3 days. Individual colonies were selected and resuspended in ddH_2_O in the ratio of 1:1 (undiluted), 1:10, 1:100, and 1:1000 (v/v). The undiluted and diluted cell samples were spread on stringent selective medium plates SD/-Leu/-Trp, SD/-Ade/-His/-Leu/-Trp, SD/-Ade/-His/-Leu/-Trp/-Met, respectively. The plates were incubated at 30°C for 5 d. The pBridge plasmid can simultaneously insert two bait proteins, with protein A, which interacts with Prey, typically inserted at the MCSI (multiple cloning site I). Upstream of MCSII, there is a Pmet25 promoter (a methionine-inducible promoter), which allows the expression of the downstream bait protein B only in the absence of methionine (Met). Therefore, by controlling the presence or absence of methionine in yeast SD culture, the impact of protein B on the interaction between protein A and Prey can be studied.

### Determination of phytohormone levels

Quantifications of IAA levels in the NSs and TCP17 transgenic *Arabidopsis* plants were carried out through liquid chromatography-tandem mass spectrometry by the Guo Cang Jian service center (Target crop). In brief, the fresh plant materials were freeze-dried in liquid nitrogen and stored at -80°C until use. The dried plant materials were powdered in a mill. 100 mg of dried leaf powders was homogenized in 1.5 ml mixed methanol: H_2_O (80:20 (v/v)) solution. The resulting extract was vortexed and ultrasound for 30 min, and then placed under 4°C for 12 h. Supernatants were collected from different samples after centrifugation, and the residues were re-extracted in 1 ml methanol through ultrasound for 30 min followed by precipitation via centrifugation. The resulting supernatants were mixed, dried through evaporation under nitrogen gas stream, and reconstituted in methanol. The solution was then filtered through a 0.22-μm filter. The samples were analyzed using a Triple Quadrupole 4500 LC/MS/MS System (AB Sciex) equipped with an ESI ion source and a Hypersil Gold C18 column (3 μm, 2.1 mm × 100 mm).

### Quantification and statistical analysis

All statistical analyses were performed by one-way ANOVA with Tukey’s test using GraphPad software or by two-sided Student’s t-test using Microsoft Excel software. Quantification analyses on protein abundance were conducted by ImageJ software.

### Accession numbers

Sequence data described in this article can be found in TAIR (www.Arabidopsis.org) under the following accession numbers: *YUC2* (AT4G13260), *YUC5* (AT5G43890), *YUC6* (AT5G25620), *YUC8* (AT4G28720), *IAA29* (AT4G32280), *IAA3* (AT1G04240), *TCP17* (AT5G08070), *SAUR22* (AT5G18050), *ACTIN2* (AT3G18780), *ACTIN5* (AT2G42170), *ACTIN8* (AT1G49240), *TCP5* (AT5G60970), *TCP8* (AT1G58100), *TCP13* (AT3G02150), *TCP22* (AT1G72010), *SlYUC5* (Solyc06g083700), *CaYUC5* (CA06g01880), *SlTCP17* (Solyc02g089020), *CaTCP17* (CA02g27290).

## Supporting information

S1 Fig The systemic Infection of TSWV in *Arabidopsis*. (A) Phenotype of TSWV-infected *Arabidopsis* plant. The photos of infected plants were taken at 12 dpi. (B) The accumulation of TSWV N protein in TSWV-infected plants determined by Western blotting. Total protein extracts were separated by SDS-PAGE and analyzed by an immunoblotting approach using an anti-N antibody. Ponceau S staining was used to estimate sample loading.
(TIF)

S2 Fig Phenotypes of NSs transgenic Arabidopsis plants. (A) Transgenic *Arabidopsis* lines expressing NSs (1 to 11) were screened and examined by RT-PCR. (B) The phenotypes of WT and NSs transgenic line #2 and line #8 *Arabidopsis* plants.
(TIF)

S3 Fig The effects of auxin phytohormone on TSWV accumulation in *N*. *benthamiana* and *Arabidopsis* plant leaves. (A) *N*. *benthamiana* plants were sprayed with DMSO or 50 μM IAA. At 3 d post treatment, phytohormone-treated leaves were inoculated again with TSWV infectious clone [L(+)opt+M(–)opt+SR(+)eGFP] via agro-infiltration. The infiltrated *N*. *benthamiana* plant leaves were harvested at 60 hpi and imaged for eGFP fluorescence loci under an inverted fluorescence microscope (Scale bars, 800 μm.). (B) Western blot assay results showing the accumulation level of eGFP at 60 hpi in the infiltrated leaves shown in (A), using anti-GFP antibody. **p < 0.01. (C) Phenotype of TSWV-inoculated *Arabidopsis* plants treated with DMSO or IAA. *Arabidopsis* plants were sprayed with DMSO and 100 μM IAA respectively. At 3 d post treatment, the fresh sap from TSWV infected tissues was mechanically inoculated onto phytohormone treated leaves. The phenotype of TSWV-inoculated plants was photographed at 12 d post inoculation. (D) TSWV accumulation was analyzed in systemic infected leaves of *Arabidopsis* plants treated with DMSO and IAA, respectively, at 12 dpi by Western blot using TSWV N specific antibodies. Immunoblot analysis of actin is used to estimate the sample loadings. * p < 0.05.
(TIF)

S4 Fig Relative expression levels of auxin response genes in WT and NSs transgenic plants. RT-qPCR analysis results showing the expressions of auxin response genes in in WT and NSs-transgenic *Arabidopsis* plants. Data are mean ± s.e.m. *P < 0.05, **P < 0.01.
(TIF)

S5 Fig NSs interacts with TCP17 full-length. Y2H assays illustrating the interaction between the NSs and TCP17 full-length or its mutants. (Upper) Schematic diagrams of the bait and the prey constructs used in Y2H assays. (Lower) The co-transformed yeast cells were grown on the SD/-T-L, SD/-T-L-H-A (lacking Trp, Leu, His and Ade) plates, respectively, for 5 d.
(TIF)

S6 Fig NSs does not interact with other TCPs. The Y2H assay results indicate that NSs cannot interact with TCP5, TCP13, TCP8, and TCP22. The co-transformed yeast cells were grown on the SD/-T-L, SD/-T-L-H-A (lacking Trp, Leu, His and Ade) plates, respectively, for 5 d.
(TIF)

S7 Fig TCP17 positively regulates auxin synthesis and signaling pathways. (A) Phenotype of *tcp17* mutant and WT *Arabidopsis* plant. (B) Relative expression levels of auxin biosynthesis genes in WT and TCP17 transgenic plants. Data are presented as mean values ± s.e.m.; n  =  3 biologically independent samples. (C) Relative expression levels of auxin response genes in WT and *tcp17* mutant *Arabidopsis* plants. Data are presented as mean values ± s.e.m.; n  =  3 biologically independent samples. *P < 0.05, **P < 0.01, ns: no significance.
(TIF)

S8 Fig TCP17 specifically activates the expression of the *YUCs* genes. (A) The mutation of the transcription binding sites are shown at the top of the image. The relative LUC activities were measured in *N*. *benthamiana* cells. The LUC/REN ratio represents the relative LUC activity. The concentration of agrobacterium individually carrying those constructs were used at OD_600_ =  1.0. The luciferase activity was assayed at 48 hpi. The luciferase activity in the treated leaves was quantified and shown in the right. Data are presented as mean values ± s.e.m.; n  =  3 biologically independent samples. (B and C) TCP17 cannot activate the expression of the *ACTIN2* and *ACTIN5* genes. Data are presented as mean values ± s.e.m.; n  =  3 biologically independent samples. ns: no significance.
(TIF)

S9 Fig The activation of *YUC5* expression by TCP17 is conserved in tomato and pepper plants. (A) Transient overexpression of SlTCP17 transcription factor activated the expression of luciferase (LUC) driven by the *SlYUC5* promoter. (B) Transient overexpression of CaTCP17 transcription factor activated the expression of luciferase (LUC) driven by the *CaYUC5* promoter. The transcription binding sites are shown at the top of the images. The relative LUC activities were measured in *N*. *benthamiana* cells. The LUC/REN ratio represents the relative LUC activity. The concentration of agrobacterium individually carrying those constructs were used at OD_600_ =  1.0. The luciferase activity was assayed at 48 hpi. The luciferase activity in the treated leaves was quantified and shown in the right. Data are presented as mean values ± s.e.m.; n  =  3 biologically independent samples. **P < 0.01, ***P < 0.001.
(TIF)

S10 Fig NSs strongly inhibit transcriptional activation of TCP17. (A-C) The relative LUC activities were measured in *N*. *benthamiana* cells. The LUC/REN ratio represents the relative LUC activity. The concentration of agrobacterium individually carrying those constructs were used at OD_600_ =  1.0. The luciferase activity was assayed at 48 hpi. The luciferase activity in the treated leaves was quantified and shown in the right. Data are presented as mean values ± s.e.m.; n  =  3 biologically independent samples. Lowercase letters a-c represent statistically different groups (one way ANOVA with Tukey’s test, p < 0.05).
(TIF)

S11 Fig Exogenous application of auxin or overexpression of YUCs can promote the expression of *DR5*. (A) Exogenous application of auxin can promote the expression of luciferase reporter gene driven by the *DR5* promoter. Relative fluorescence signal intensity of each treatment in the left was quantified and shown in the right. Data are presented as mean values ± s.e.m. (B) Overexpression of *YUCs* can promote the expression of luciferase reporter gene driven by the *DR5* promoter. Relative fluorescence signal intensity of each treatment in the left was quantified and shown in the right. Data are presented as mean values ± s.e.m.; n  =  3 biologically independent samples. Lowercase letters a-d represent statistically different groups (one way ANOVA with Tukey’s test, p < 0.05).
(TIF)

S12 Fig NSs have no effect on DNA binding Ability of TCP17. (A) EMSA showing the in vitro binding of recombinant TCP17 to the promoters of *YUC5*. The wild type (WT) and mutant probe sequences are shown at the top of the image. BSA (negative control) or TCP17 was incubated with probe, followed by separation on native agarose gel. (B) The effect of NSs on DNA binding ability of TCP17.
(TIF)

S13 Fig The NSs mutant that does not interact with TCP17 reduces the diminishes the infectivity of TSWV. (A) Interaction surface of NSs bound to TCP17. The protein interaction surface and residues are predicted by AlphaFold3. The distances of the crosslinking residues between NSs and TCP17 are marked. The purple protein structure model is NSs, and the green protein structure model is TCP17. (B) The Y2H assay results show that the NSs with mutated interacting residues is unable to interact with TCP17. The mutation sites of NSs are shown at the top of the image. The yeast co-transformed with BD- and AD-derivative constructs was plated on SD/-L-T-H-A and SD/-L-T. (C) Effects of NSs mutant on TSWV infection. TSWV infectious clone was agroinfiltrated into *N*. *benthamiana* together with NSs or NSs (mut). The GFP fluorescence indicates TSWV -infected cells and was photographed by confocal microscope at 48 hpi. Bars, 50 μm. (D) Western blot assay results showing the accumulation level of GFP at 48 hpi in the infiltrated leaves shown in (C), using anti-GFP antibody. Ponceau S staining was used to estimate sample loading.
(TIF)

S1 Table Primers used in this study. (XLSX)

S2 Table Source data for graphs in this study. (XLSX)

S1 Data Original western blot images. (PDF)
