# Supplementary figures and images for: Different viral effectors hijack TCP17, a key transcription factor for host Auxin synthesis, to promote viral infection
Source: PLoS Pathog. 2024 Aug 29;20(8):e1012510. doi: 10.1371/journal.ppat.1012510 (PMC11389919; doi:10.1371/journal.ppat.1012510)

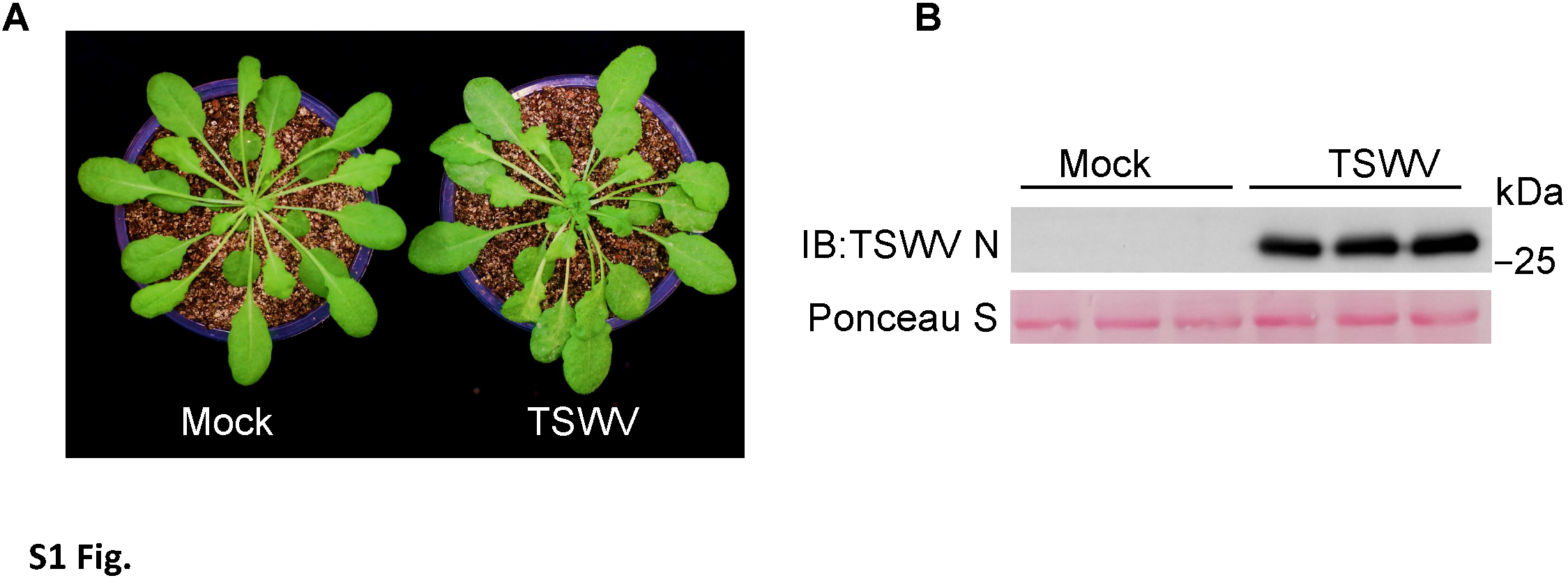

Supplement: S1 Fig — (A) Phenotype of TSWV-infected Arabidopsis plant. The photos of infected plants were taken at 12 dpi. (B) The accumulation of TSWV N protein in TSWV-infected plants determined by Western blotting. Total protein extracts were separated by SDS-PAGE and analyzed by an immunoblotting approach using an anti-N antibody. Ponceau S staining was used to estimate sample loading. (TIF) [file ppat.1012510.s001.tif]

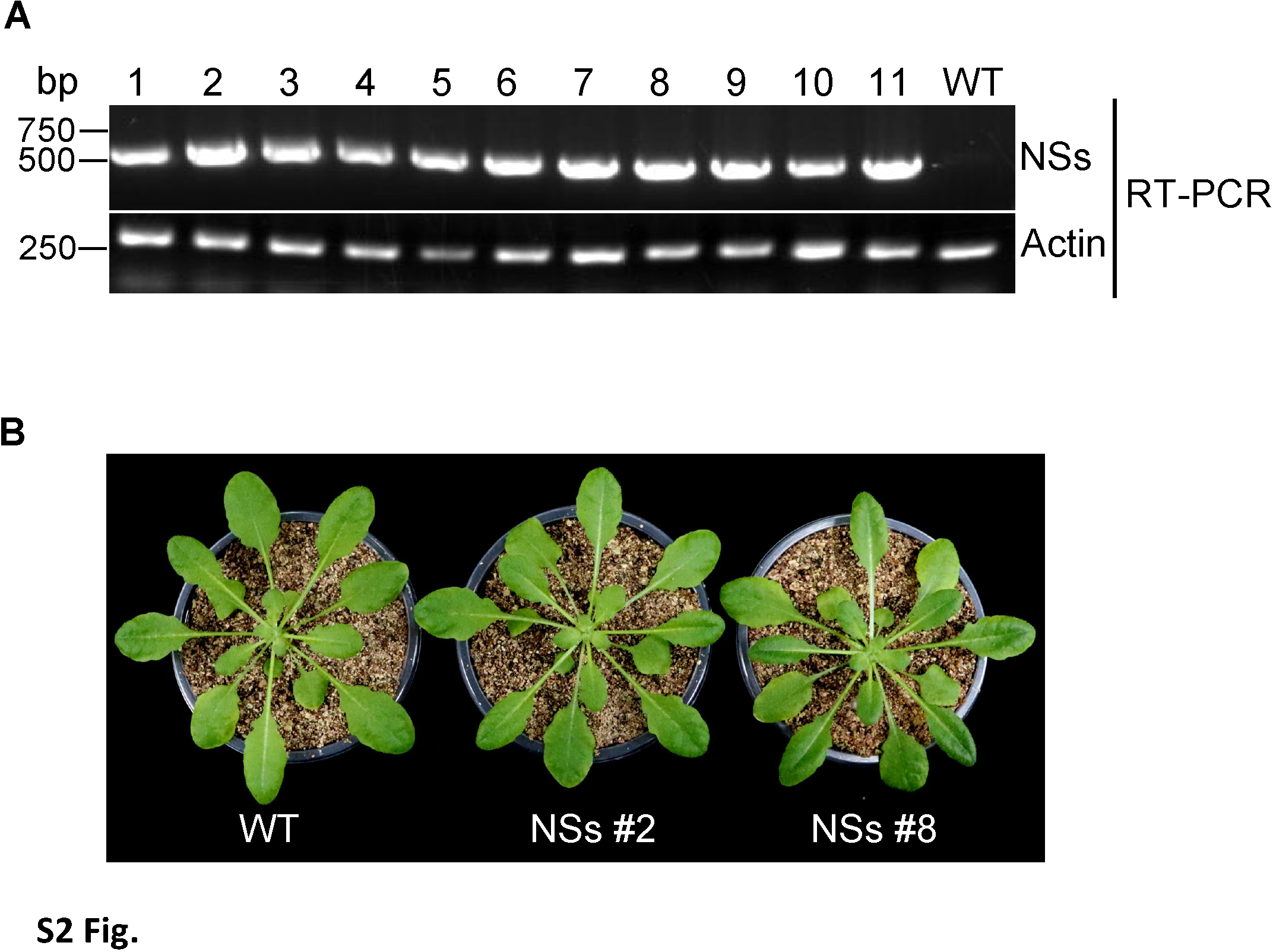

Supplement: S2 Fig — (A) Transgenic Arabidopsis lines expressing NSs (1 to 11) were screened and examined by RT-PCR. (B) The phenotypes of WT and NSs transgenic line #2 and line #8 Arabidopsis plants. (TIF) [file ppat.1012510.s002.tif]

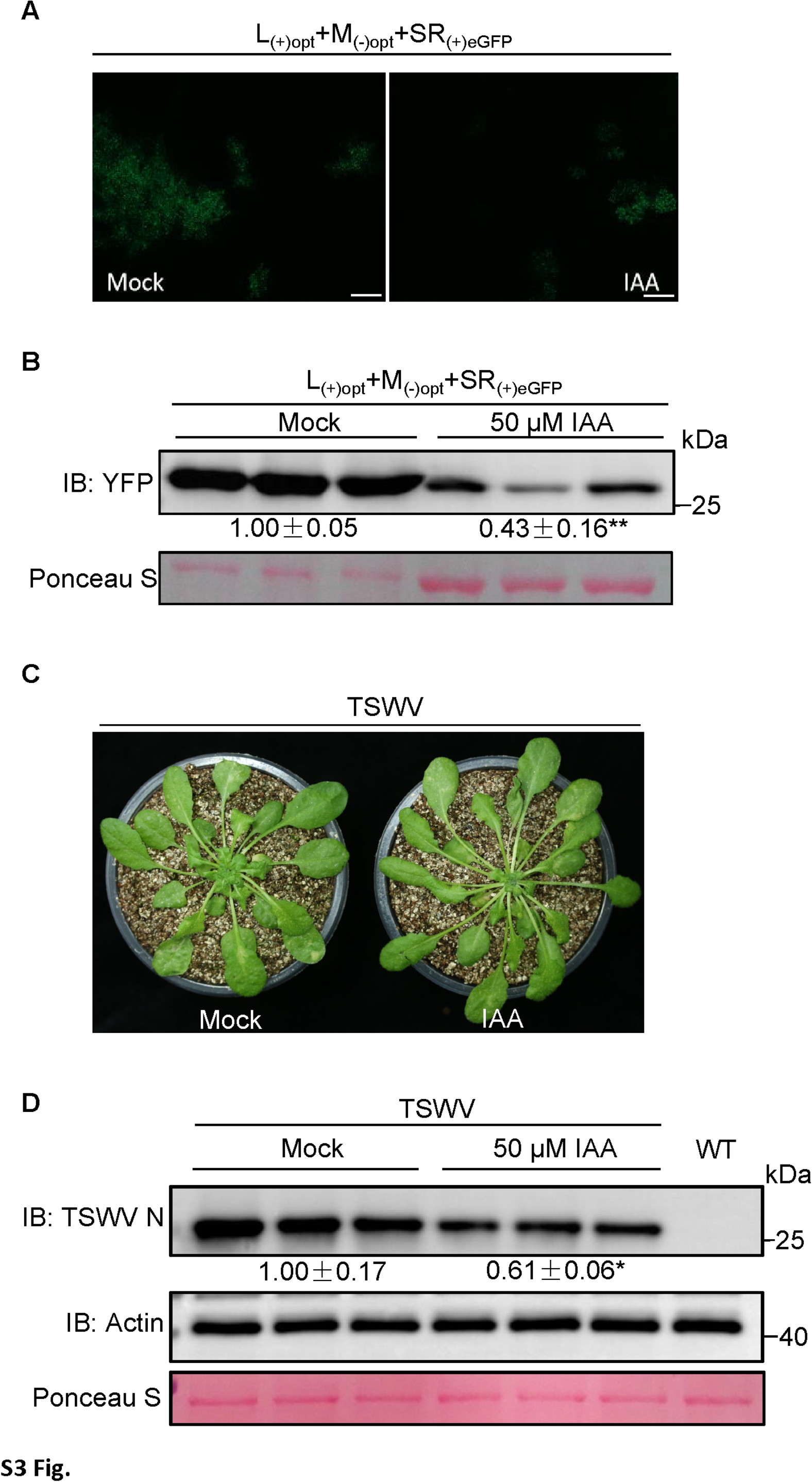

Supplement: S3 Fig — (A) N. benthamiana plants were sprayed with DMSO or 50 μM IAA. At 3 d post treatment, phytohormone-treated leaves were inoculated again with TSWV infectious clone [L(+)opt+M(–)opt+SR(+)eGFP] via agro-infiltration. The infiltrated N. benthamiana plant leaves were harvested at 60 hpi and imaged for eGFP fluorescence loci under an inverted fluorescence microscope (Scale bars, 800 μm.). (B) Western blot assay results showing the accumulation level of eGFP at 60 hpi in the infiltrated leaves shown in (A), using anti-GFP antibody. **p < 0.01. (C) Phenotype of TSWV-inoculated Arabidopsis plants treated with DMSO or IAA. Arabidopsis plants were sprayed with DMSO and 100 μM IAA respectively. At 3 d post treatment, the fresh sap from TSWV infected tissues was mechanically inoculated onto phytohormone treated leaves. The phenotype of TSWV-inoculated plants was photographed at 12 d post inoculation. (D) TSWV accumulation was analyzed in systemic infected leaves of Arabidopsis plants treated with DMSO and IAA, respectively, at 12 dpi by Western blot using TSWV N specific antibodies. Immunoblot analysis of actin is used to estimate the sample loadings. * p < 0.05. (TIF) [file ppat.1012510.s003.tif]

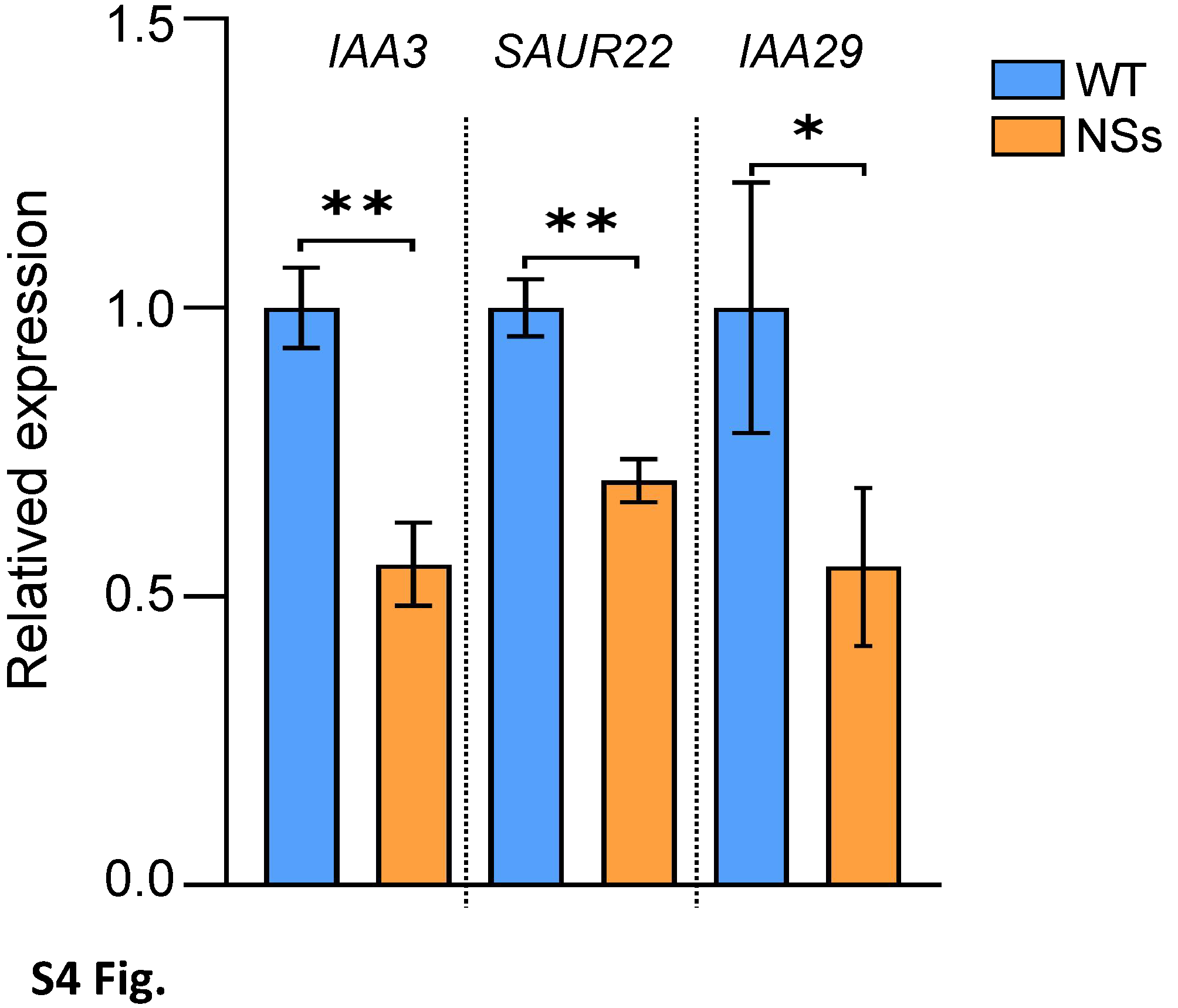

Supplement: S4 Fig — RT-qPCR analysis results showing the expressions of auxin response genes in in WT and NSs-transgenic Arabidopsis plants. Data are mean ± s.e.m. *P < 0.05, **P < 0.01. (TIF) [file ppat.1012510.s004.tif]

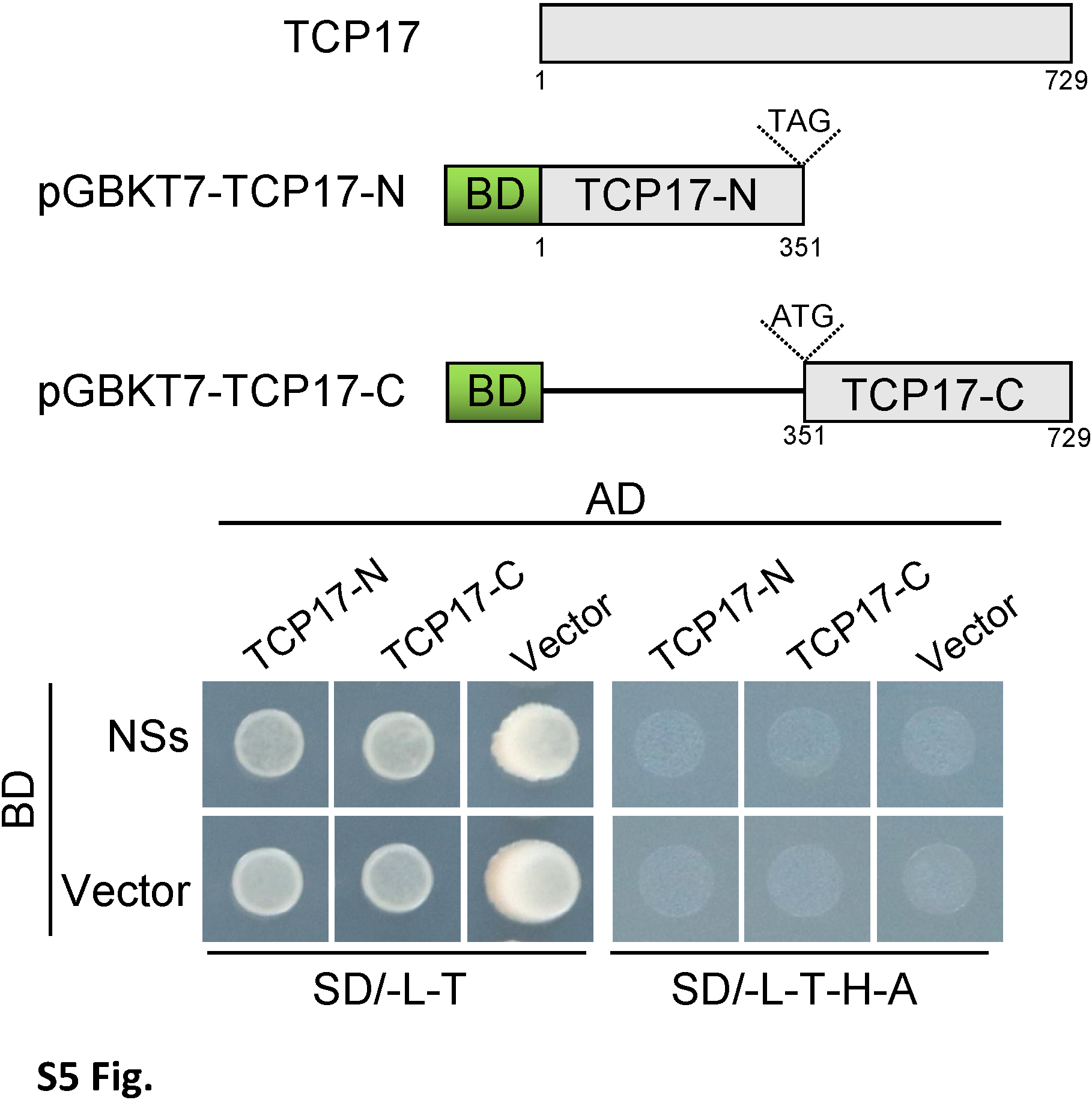

Supplement: S5 Fig — Y2H assays illustrating the interaction between the NSs and TCP17 full-length or its mutants. (Upper) Schematic diagrams of the bait and the prey constructs used in Y2H assays. (Lower) The co-transformed yeast cells were grown on the SD/-T-L, SD/-T-L-H-A (lacking Trp, Leu, His and Ade) plates, respectively, for 5 d. (TIF) [file ppat.1012510.s005.tif]

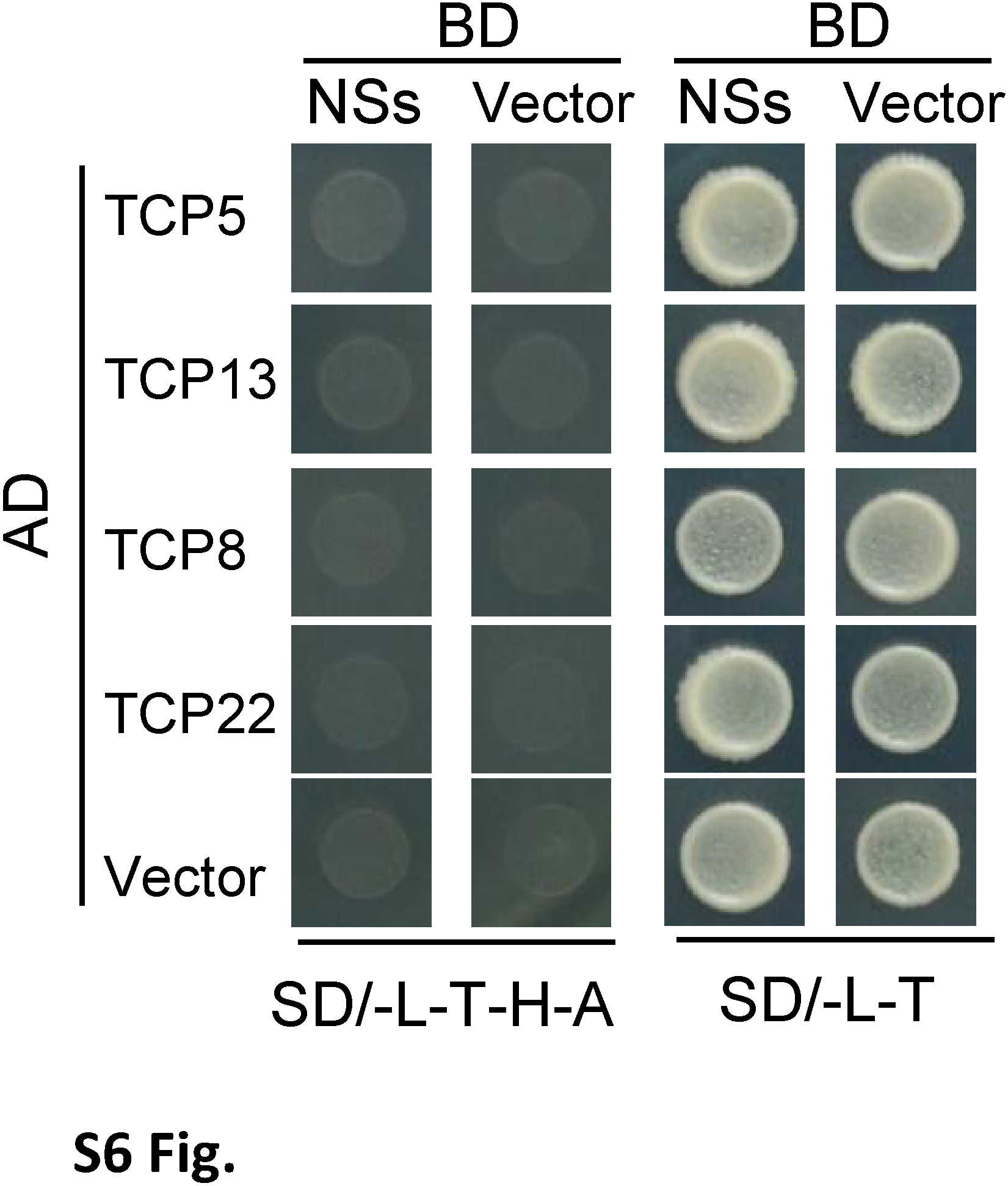

Supplement: S6 Fig — The Y2H assay results indicate that NSs cannot interact with TCP5, TCP13, TCP8, and TCP22. The co-transformed yeast cells were grown on the SD/-T-L, SD/-T-L-H-A (lacking Trp, Leu, His and Ade) plates, respectively, for 5 d. (TIF) [file ppat.1012510.s006.tif]

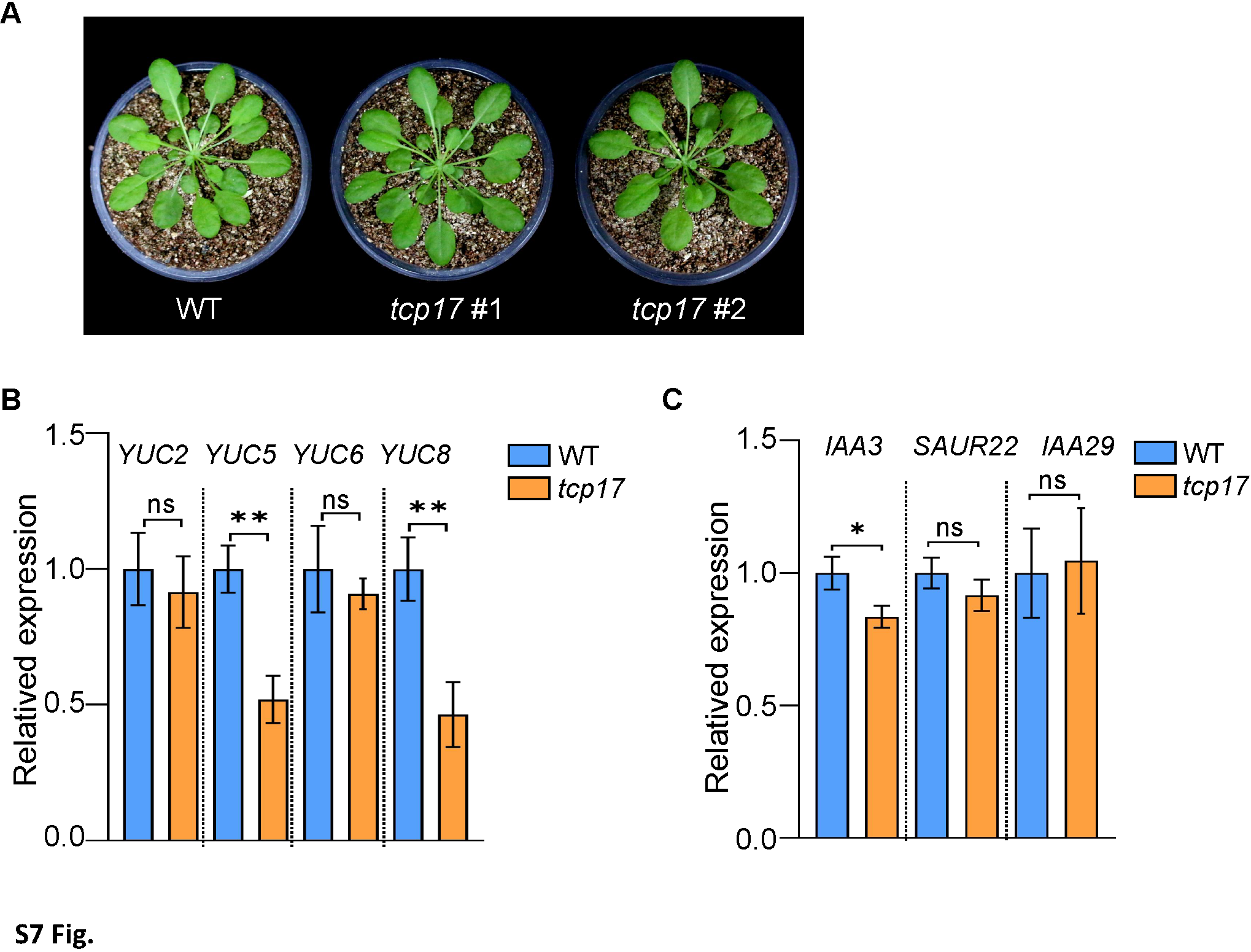

Supplement: S7 Fig — (A) Phenotype of tcp17 mutant and WT Arabidopsis plant. (B) Relative expression levels of auxin biosynthesis genes in WT and TCP17 transgenic plants. Data are presented as mean values ± s.e.m.; n = 3 biologically independent samples. (C) Relative expression levels of auxin response genes in WT and tcp17 mutant Arabidopsis plants. Data are presented as mean values ± s.e.m.; n = 3 biologically independent samples. *P < 0.05, **P < 0.01, ns: no significance. (TIF) [file ppat.1012510.s007.tif]

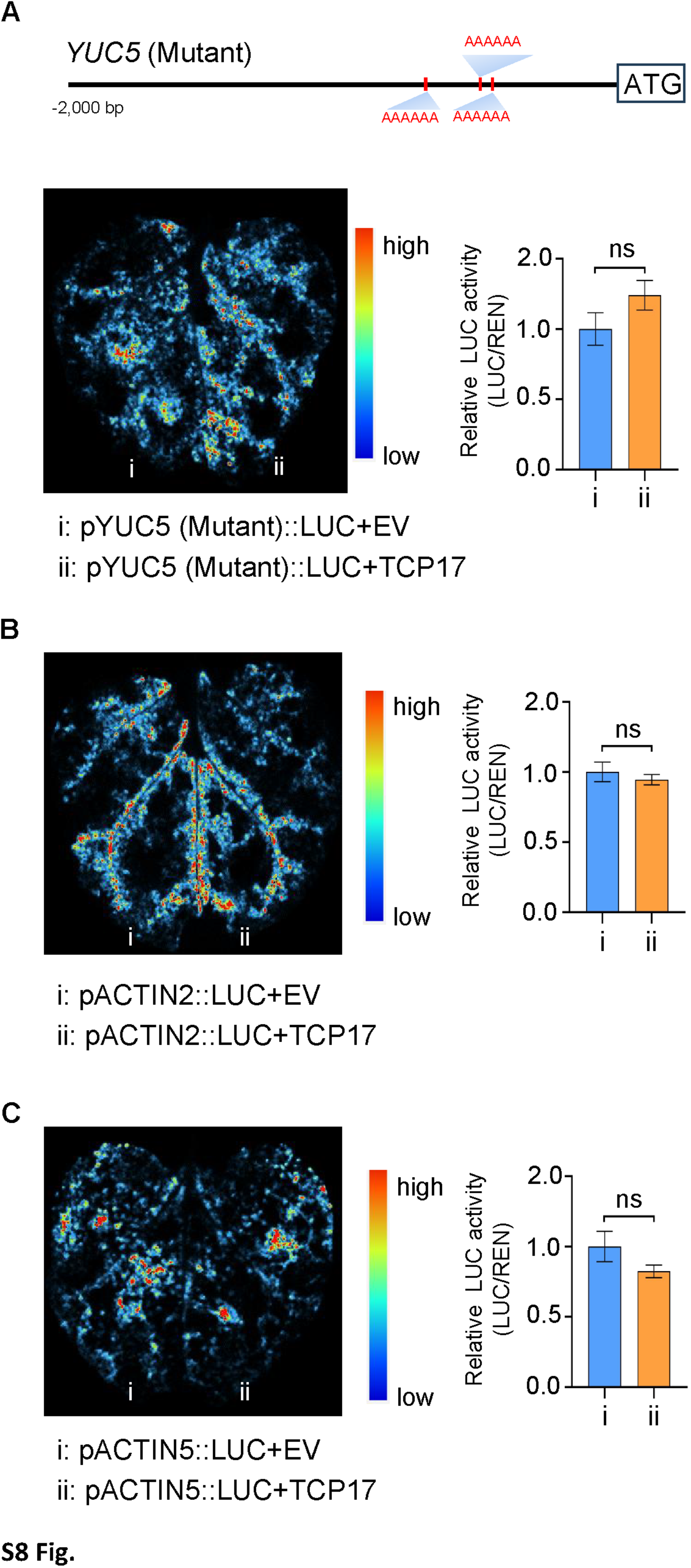

Supplement: S8 Fig — (A) The mutation of the transcription binding sites are shown at the top of the image. The relative LUC activities were measured in N. benthamiana cells. The LUC/REN ratio represents the relative LUC activity. The concentration of agrobacterium individually carrying those constructs were used at OD600 = 1.0. The luciferase activity was assayed at 48 hpi. The luciferase activity in the treated leaves was quantified and shown in the right. Data are presented as mean values ± s.e.m.; n = 3 biologically independent samples. (B and C) TCP17 cannot activate the expression of the ACTIN2 and ACTIN5 genes. Data are presented as mean values ± s.e.m.; n = 3 biologically independent samples. ns: no significance. (TIF) [file ppat.1012510.s008.tif]

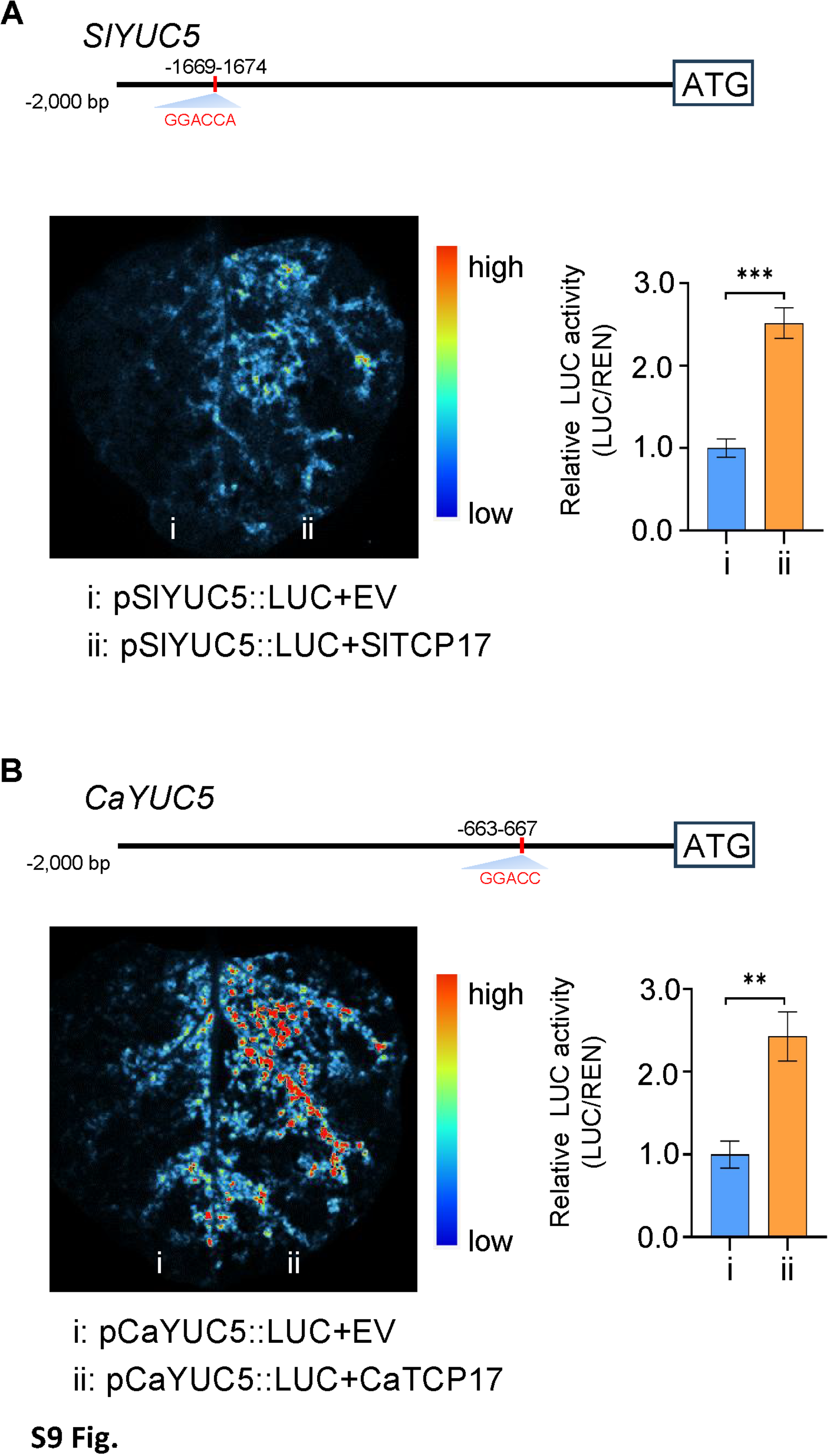

Supplement: S9 Fig — (A) Transient overexpression of SlTCP17 transcription factor activated the expression of luciferase (LUC) driven by the SlYUC5 promoter. (B) Transient overexpression of CaTCP17 transcription factor activated the expression of luciferase (LUC) driven by the CaYUC5 promoter. The transcription binding sites are shown at the top of the images. The relative LUC activities were measured in N. benthamiana cells. The LUC/REN ratio represents the relative LUC activity. The concentration of agrobacterium individually carrying those constructs were used at OD600 = 1.0. The luciferase activity was assayed at 48 hpi. The luciferase activity in the treated leaves was quantified and shown in the right. Data are presented as mean values ± s.e.m.; n = 3 biologically independent samples. **P < 0.01, ***P < 0.001. (TIF) [file ppat.1012510.s009.tif]

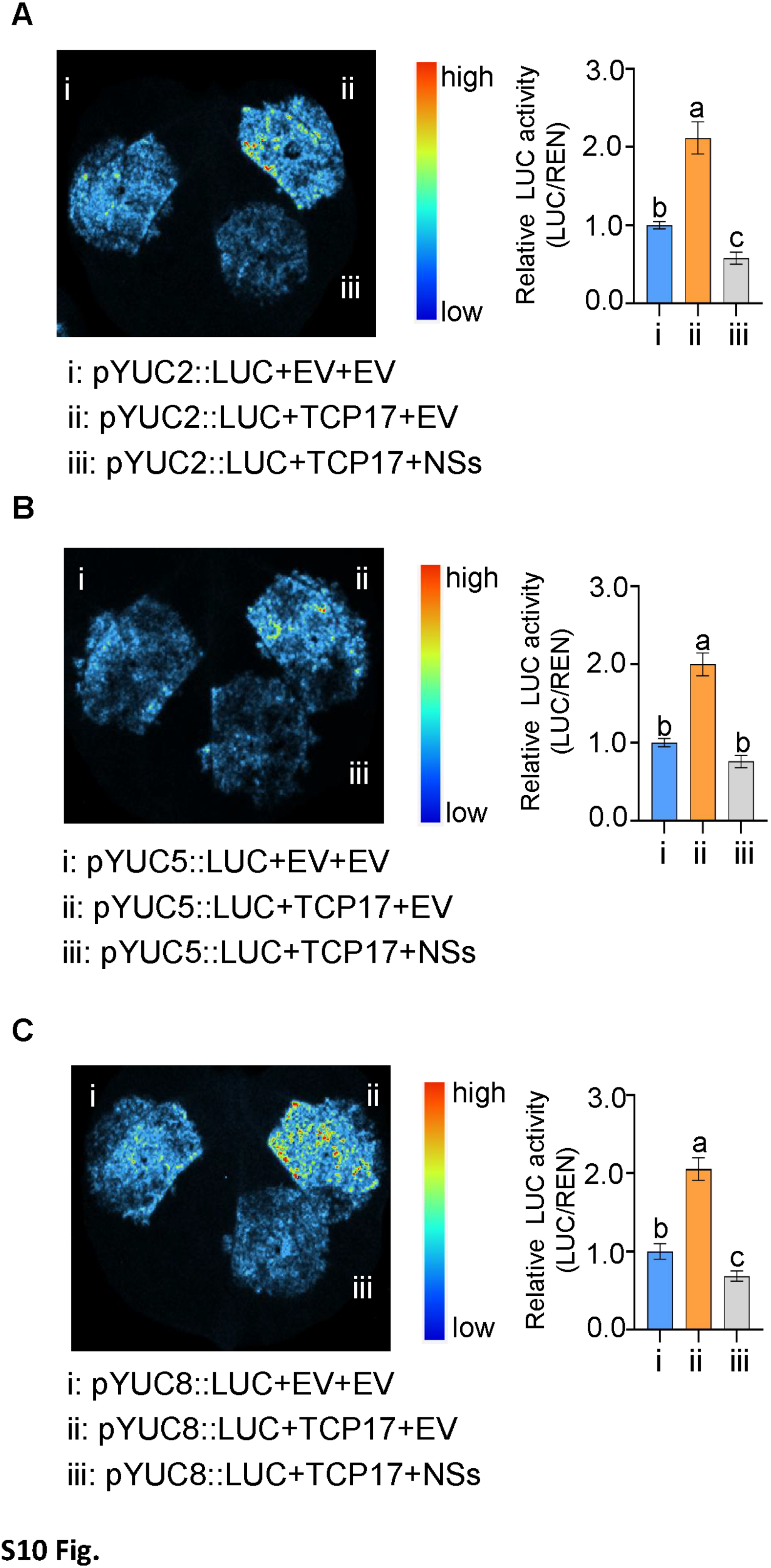

Supplement: S10 Fig — (A-C) The relative LUC activities were measured in N. benthamiana cells. The LUC/REN ratio represents the relative LUC activity. The concentration of agrobacterium individually carrying those constructs were used at OD600 = 1.0. The luciferase activity was assayed at 48 hpi. The luciferase activity in the treated leaves was quantified and shown in the right. Data are presented as mean values ± s.e.m.; n = 3 biologically independent samples. Lowercase letters a-c represent statistically different groups (one way ANOVA with Tukey’s test, p < 0.05). (TIF) [file ppat.1012510.s010.tif]

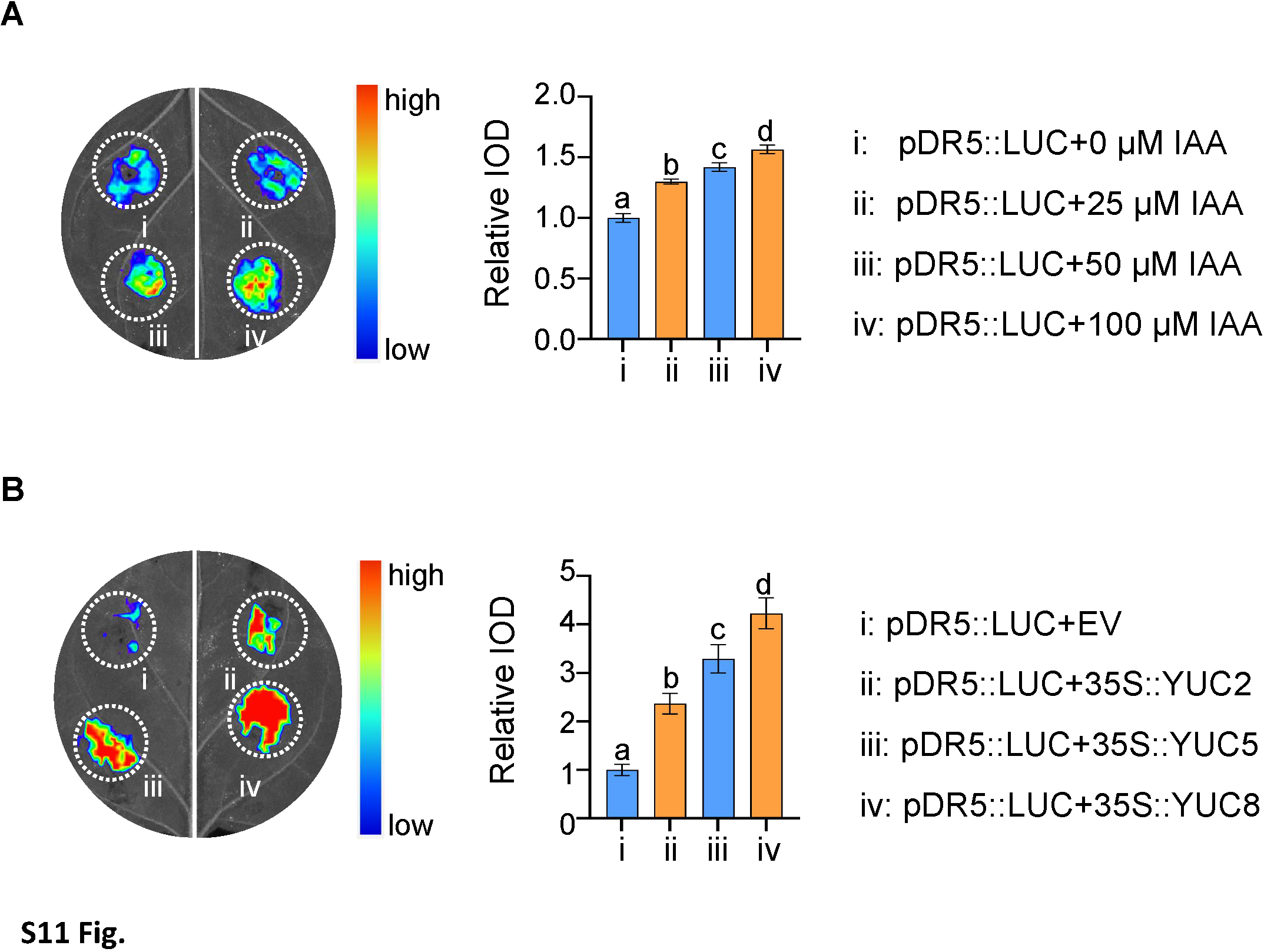

Supplement: S11 Fig — (A) Exogenous application of auxin can promote the expression of luciferase reporter gene driven by the DR5 promoter. Relative fluorescence signal intensity of each treatment in the left was quantified and shown in the right. Data are presented as mean values ± s.e.m. (B) Overexpression of YUCs can promote the expression of luciferase reporter gene driven by the DR5 promoter. Relative fluorescence signal intensity of each treatment in the left was quantified and shown in the right. Data are presented as mean values ± s.e.m.; n = 3 biologically independent samples. Lowercase letters a-d represent statistically different groups (one way ANOVA with Tukey’s test, p < 0.05). (TIF) [file ppat.1012510.s011.tif]

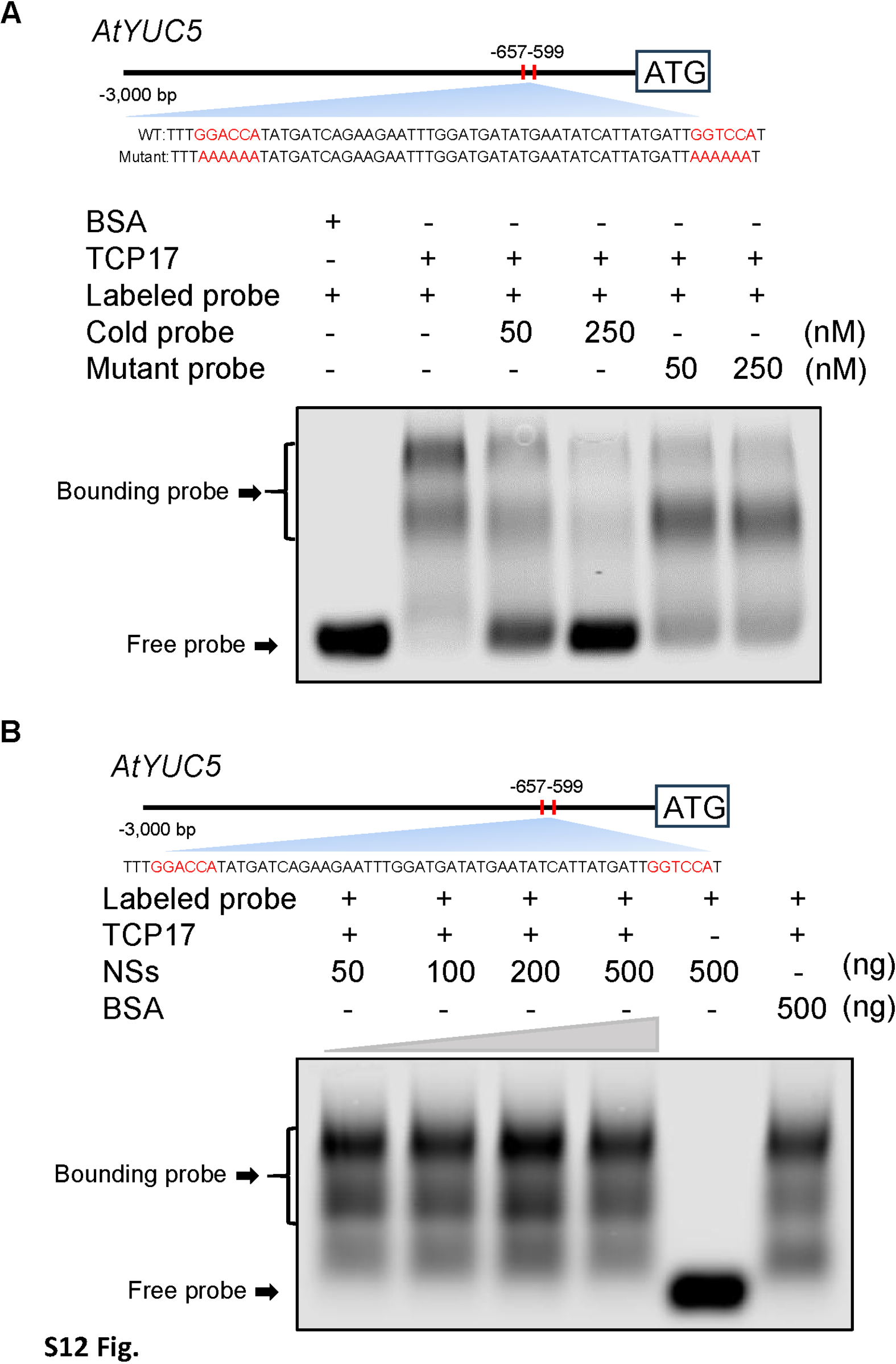

Supplement: S12 Fig — (A) EMSA showing the in vitro binding of recombinant TCP17 to the promoters of YUC5. The wild type (WT) and mutant probe sequences are shown at the top of the image. BSA (negative control) or TCP17 was incubated with probe, followed by separation on native agarose gel. (B) The effect of NSs on DNA binding ability of TCP17. (TIF) [file ppat.1012510.s012.tif]

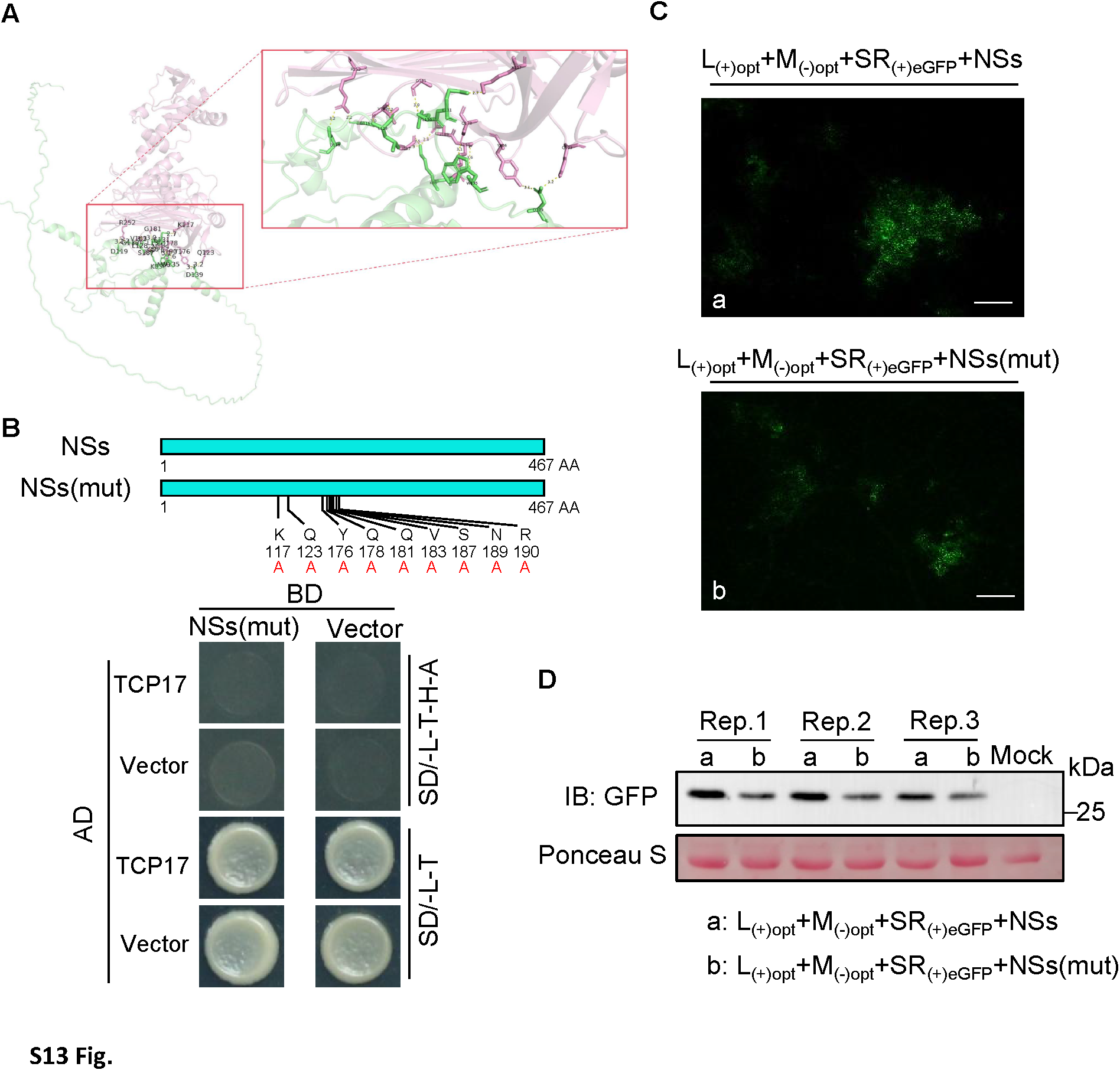

Supplement: S13 Fig — (A) Interaction surface of NSs bound to TCP17. The protein interaction surface and residues are predicted by AlphaFold3. The distances of the crosslinking residues between NSs and TCP17 are marked. The purple protein structure model is NSs, and the green protein structure model is TCP17. (B) The Y2H assay results show that the NSs with mutated interacting residues is unable to interact with TCP17. The mutation sites of NSs are shown at the top of the image. The yeast co-transformed with BD- and AD-derivative constructs was plated on SD/-L-T-H-A and SD/-L-T. (C) Effects of NSs mutant on TSWV infection. TSWV infectious clone was agroinfiltrated into N. benthamiana together with NSs or NSs (mut). The GFP fluorescence indicates TSWV -infected cells and was photographed by confocal microscope at 48 hpi. Bars, 50 μm. (D) Western blot assay results showing the accumulation level of GFP at 48 hpi in the infiltrated leaves shown in (C), using anti-GFP antibody. Ponceau S staining was used to estimate sample loading. (TIF) [file ppat.1012510.s013.tif]

**Fig. 2B**

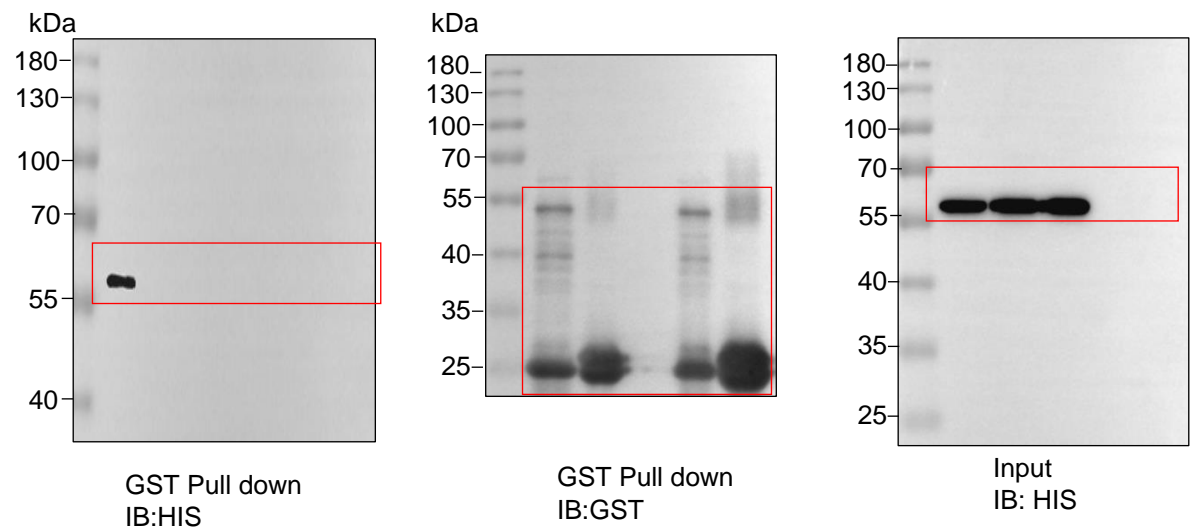

**Fig. 2C**

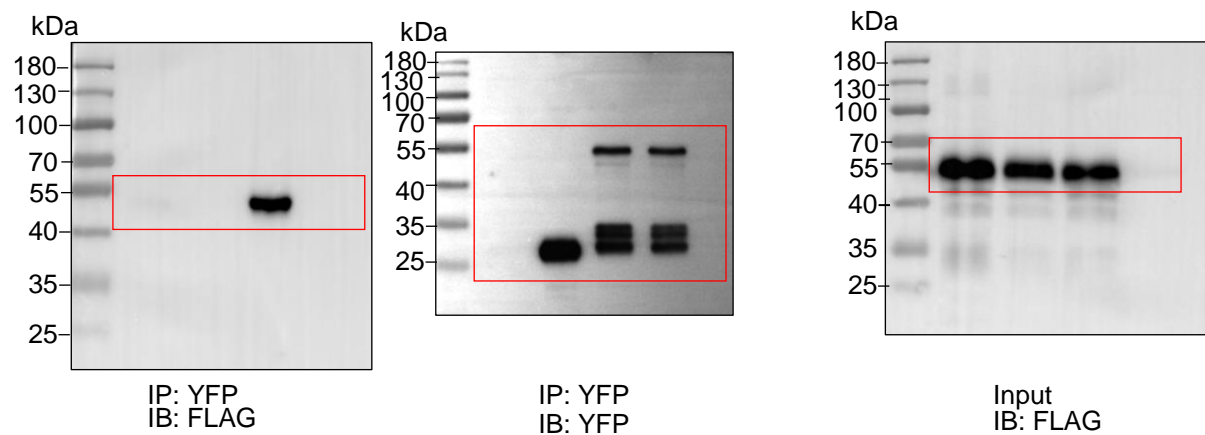

**Fig. 3F**

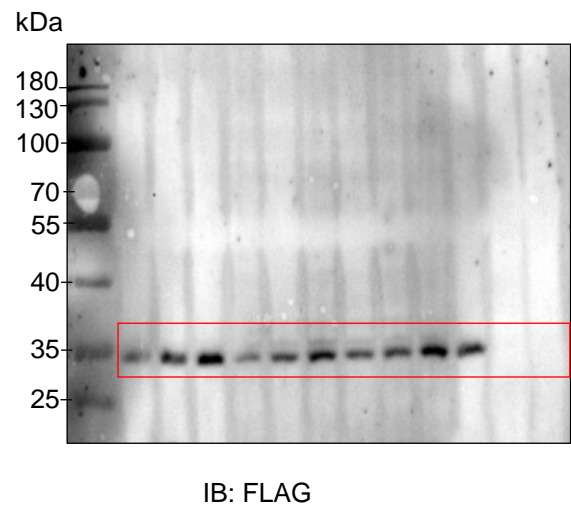

**Fig. 4B**

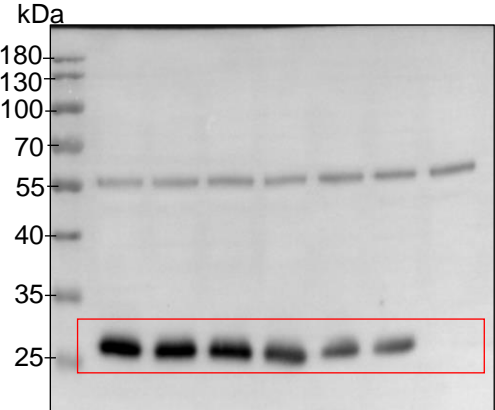

IB:TSWV N

**Fig. 4D**

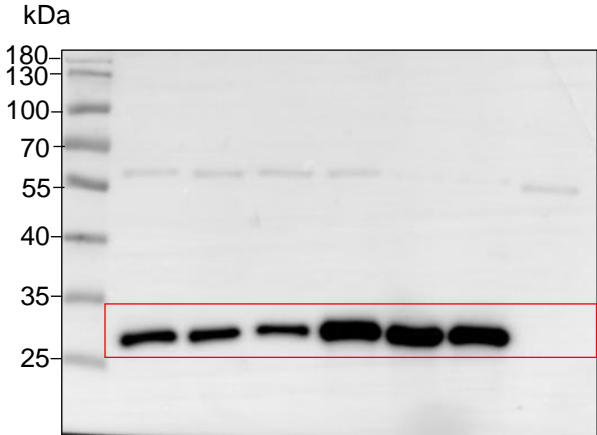

IB:TSWV N

**Fig. 6B**

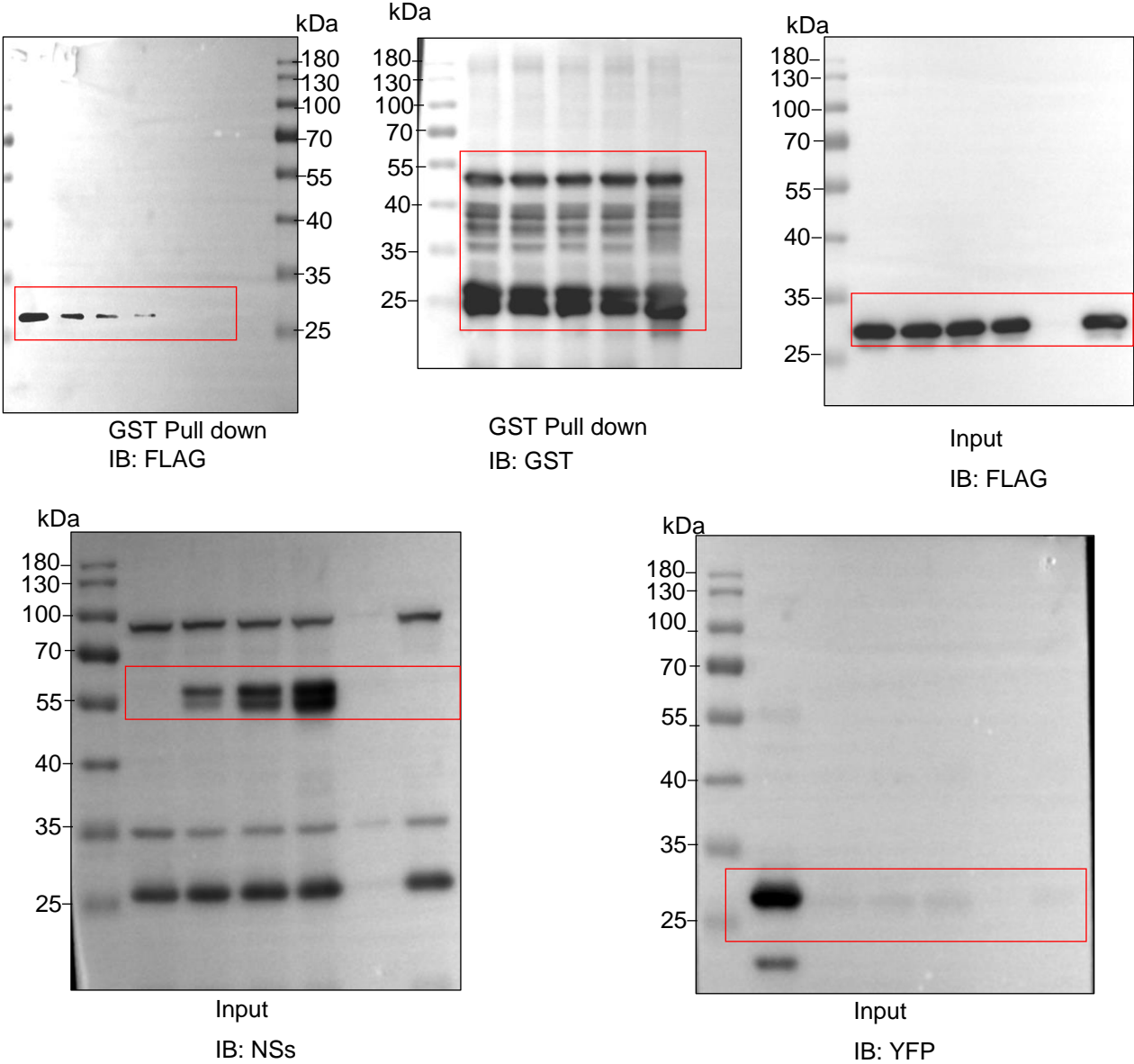

**Fig. 7D**

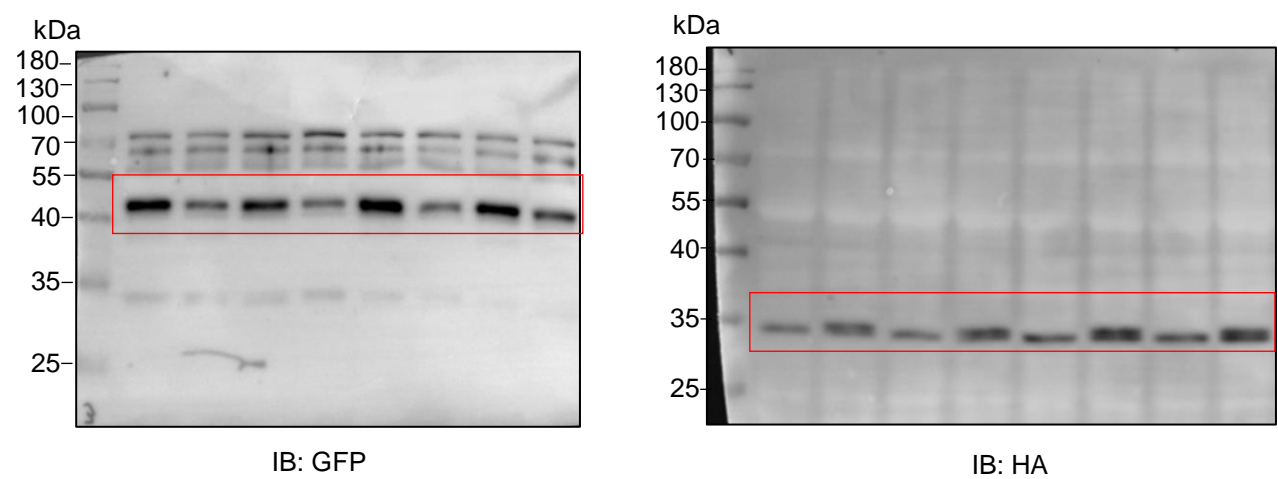

**Fig. S1B**

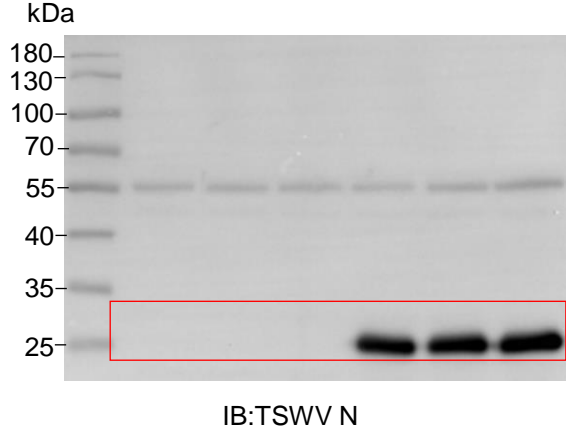

**Fig. S3B**

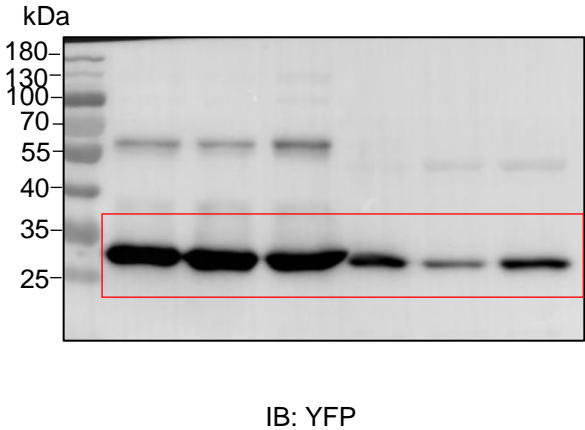

**Fig. S3B**

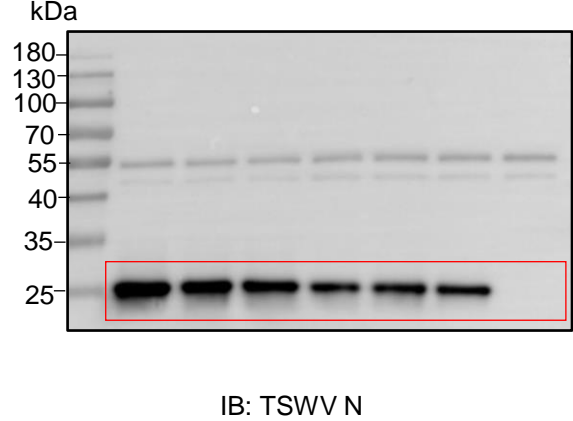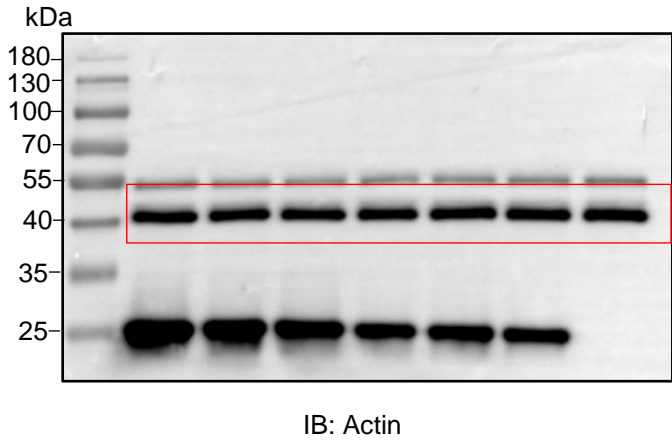

**Fig. S13D**

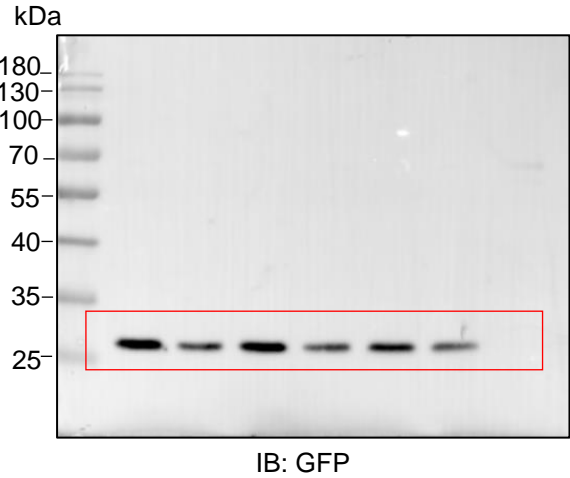

Supplement: S1 Data — (PDF) [file ppat.1012510.s016.pdf]
